# Whole exome sequencing of patients with varicella-zoster virus and herpes simplex virus induced acute retinal necrosis reveals rare disease-associated genetic variants

**DOI:** 10.3389/fnmol.2023.1253040

**Published:** 2023-10-25

**Authors:** Johanna L. Heinz, Sigrid M. A. Swagemakers, Joanna von Hofsten, Marie Helleberg, Michelle M. Thomsen, Kerstin De Keukeleere, Joke H. de Boer, Tomas Ilginis, Georges M. G. M. Verjans, Peter M. van Hagen, Peter J. van der Spek, Trine H. Mogensen

**Affiliations:** ^1^Department of Biomedicine, Aarhus University, Aarhus, Denmark; ^2^Department of Infectious Diseases, Aarhus University Hospital, Aarhus, Denmark; ^3^Department of Pathology and Clinical Bioinformatics, Erasmus University Medical Center, Rotterdam, Netherlands; ^4^Department of Clinical Neuroscience, Institute of Neuroscience and Physiology, Sahlgrenska Academy, University of Gothenburg, Gothenburg, Sweden; ^5^Department of Ophthalmology, Halland Hospital Halmstad, Halmstad, Sweden; ^6^Department of Infectious Diseases, Rigshospitalet, Copenhagen University Hospital, Copenhagen, Denmark; ^7^Center of Excellence for Health, Immunity and Infections, Rigshospitalet, Copenhagen University Hospital, Copenhagen, Denmark; ^8^Department of Ophthalmology, University Medical Centre Utrecht, Utrecht, Netherlands; ^9^Department of Ophthalmology, Rigshospitalet, Copenhagen University Hospital, Copenhagen, Denmark; ^10^HerpeslabNL, Department of Viroscience, Erasmus University Medical Center, Rotterdam, Netherlands; ^11^Department of Internal Medicine and Immunology, Erasmus University Medical Center, Rotterdam, Netherlands

**Keywords:** acute retinal necrosis (ARN), interferon, autophagy, apoptosis, whole exome sequencing

## Abstract

**Purpose:**

Herpes simplex virus (HSV) and varicella-zoster virus (VZV) are neurotropic human alphaherpesviruses endemic worldwide. Upon primary infection, both viruses establish lifelong latency in neurons and reactivate intermittently to cause a variety of mild to severe diseases. Acute retinal necrosis (ARN) is a rare, sight-threatening eye disease induced by ocular VZV or HSV infection. The virus and host factors involved in ARN pathogenesis remain incompletely described. We hypothesize an underlying genetic defect in at least part of ARN cases.

**Methods:**

We collected blood from 17 patients with HSV-or VZV-induced ARN, isolated DNA and performed Whole Exome Sequencing by Illumina followed by analysis in Varseq with criteria of CADD score > 15 and frequency in GnomAD < 0.1% combined with biological filters. Gene modifications relative to healthy control genomes were filtered according to high quality and read-depth, low frequency, high deleteriousness predictions and biological relevance.

**Results:**

We identified a total of 50 potentially disease-causing genetic variants, including missense, frameshift and splice site variants and on in-frame deletion in 16 of the 17 patients. The vast majority of these genes are involved in innate immunity, followed by adaptive immunity, autophagy, and apoptosis; in several instances variants within a given gene or pathway was identified in several patients.

**Discussion:**

We propose that the identified variants may contribute to insufficient viral control and increased necrosis ocular disease presentation in the patients and serve as a knowledge base and starting point for the development of improved diagnostic, prophylactic, and therapeutic applications.

## Introduction

Varicella-zoster virus (VZV) and herpes simplex virus (HSV)-1 and -2 are double-stranded (ds) DNA alphaherpesviruses which infect most individuals in a native community (Fields et al., 2013d), usually, leading to rather mild disease. VZV is most commonly acquired in early childhood during which primary infection gives rise to varicella (chickenpox). Subsequently, VZV becomes latent in dorsal root sensory neurons and cranial nerves and often reactivates later in life, then causing herpes zoster (shingles) (Fields et al., 2013c). Similarly, HSV becomes latent after primary infection in sensory ganglia; HSV-1 typically resides within trigeminal ganglia, HSV-2 within lumbar-and sacral ganglia. Classically, both primary infection and reactivation is followed by cold sores or genital herpes (Fields et al., 2013b). In rare cases, infection with both viruses may spread to the central nervous system (CNS), causing severe neurological disease, such as meningitis or encephalitis (Kleinschmidt-DeMasters and Gilden, 2001; Tyler, 2004; Alvarez et al., 2020).

Involvement of the ocular sensory nerves during alphaherpesvirus infection may lead to acute retinal necrosis (ARN), a rare and sight-threatening inflammation of the posterior part of the eye affecting various layers of the uvea, blood vessels, and full thickness necrosis of the retinal neuroepithelium, as described by the American Uveitis Society in 1994 (Holland, 1994). The exact molecular mechanism by which viral reactivation might lead to ARN onset and whether this pathway is the same for the different alphaherpesviruses remains incompletely understood. The primary site of latency most likely is the trigeminal ganglion with the virus spreading through the 1. branch of the trigeminal nerve, although reactivation from other sites may also be envisaged. ARN is a rare disease manifestation, the prevalence only amounting to 1 in 2 million individuals per year (Muthiah et al., 2007). The necrosis of affected tissues leads to various degrees of visual symptoms, including potentially permanent loss of central vision or the visual field due to retinal detachment, optic nerve or macula damage. In the absence of treatment the condition progresses rapidly and often with disadvantageous visual outcome in one or even both eyes. Unfortunately, even with systemic antiviral treatment the prognosis remains poor, with detachment of the retina as the most common complication (Lau et al., 2007; Hillenkamp et al., 2009; Iwahashi-Shima et al., 2013). VZV infection is most commonly associated with ARN, followed by HSV-1 and HSV-2 (Lau et al., 2007; Grahn and Studahl, 2015; Cox and Miller, 2022). Since many patients have reached adulthood before disease onset, it is reasonable to assume that ARN is most often a complication of reactivation rather than primary infection. Importantly, herpes simplex encephalitis (HSE) appears to be a risk factor for developing ARN later in life, highlighting the connections between the two phenotypes (Ahmadieh et al., 1991; Levinson et al., 1999; Ganatra et al., 2000; Maertzdorf et al., 2001; von Hofsten et al., 2019) and the origin of the retina as a neuroepithelium.

CNS infection by alphaherpesviruses preferentially affects individuals of the extremes of age, children and the elderly, as well as the immunocompromised (Whitley and Gnann, 2002; Wiegering et al., 2011; Fields et al., 2013a). However, occurrence in young, otherwise healthy individuals suggests a role for specific, so far undescribed inborn errors of immunity (IEI) (Kennedy and Mogensen, 2020; Herlin et al., 2021). Differences in virulence between different viral strains have also been suggested as a determinant of disease severity (Richards et al., 1981; Kolb et al., 2016; Pandey et al., 2017; Shipley et al., 2018; Pandey and Szpara, 2019). However, these observations cannot explain all interindividual variability in disease severity, thus implicating a major role of the host immune response in at least part of the cases (Brandt, 2005; Thompson et al., 2014; Kennedy and Mogensen, 2020). Clinical VZV isolates exhibit only minor sequence variation, which so far has not been conclusively connected to disease outcome in humans (Breuer, 2010). In a case of VZV ARN only minor sequence diversity in the viral genome was described (von Hofsten et al., 2020).

Numerous IEI have been described to cause severe disease after infection with VZV, HSV or other viral pathogens. Most strikingly, individuals with severe combined immunodeficiency (SCID) or natural killer (NK) deficiency are especially vulnerable to potentially fatal infection with a wide range of pathogens (Biron et al., 1989; Brooks et al., 1990). In addition, a number of single gene IEI have been demonstrated to underly increased susceptibility to severe viral infections, including mutations in *GATA2* (Mace et al., 2013), *DOCK2* (Dobbs et al., 2015), *DOCK8* (Zhang et al., 2009), *IFNGR1* (Roesler et al., 1999), *TYK2* (Kreins et al., 2015), *STAT1* (Dupuis et al., 2003), *CORA1A* (Yee et al., 2016), *MCM4* (Gineau et al., 2012), and *STK4* (Abdollahpour et al., 2012; Jørgensen et al., 2021). Moreover, IEI may also lead to a more narrow disease phenotypes, as in the case of HSE with disease causing mutations identified in *TLR3* (Zhang et al., 2007; Mørk et al., 2015) and genes encoding downstream signaling molecules *UNC93B* (Casrouge et al., 2006), *TRIF* (Sancho-Shimizu et al., 2011), *TRAF3* (Pérez de Diego et al., 2010), *TBK1* (Herman et al., 2012), *IRF3* (Andersen et al., 2015), and *IFNAR1* (Bastard et al., 2021). Of note, a mutation in *TLR3* has also been described in a patient with recurrent herpes zoster ophthalmicus (Liang et al., 2020). In addition to these interferon (IFN)-related genes, IEI affecting genes encoding for the RNA debranching molecule *DBR1* (Zhang et al., 2018) as well as for *SNORA31* (Lafaille et al., 2019) have been identified. More recently, mutations in genes involved in the autophagy pathway, *MAP1LC3B2* and *ATG4*, have been shown to predispose to recurrent HSV-2 lymphocytic Mollaret meningitis (Hait et al., 2020). Finally, single gene IEI in different subunits of RNA polymerase III have been identified first in children with severe acute VZV CNS or lung infection upon primary infection (Ogunjimi et al., 2017) and subsequently in otherwise healthy adults upon VZV reactivation (Carter-Timofte et al., 2018a, 2019). This collection of single gene IEIs in severe disease after viral infection suggest potentially similar role in ARN pathogenesis.

Despite the current literature and findings on IEI and associated alpha-herpesvirus infections of the CNS, the underlying genetic etiology underlying of a large fraction of the severely affected patients remains unexplained. Here, we describe potentially disease-causing variants in genes encoding molecules regulating innate and adaptive immune responses, apoptosis and autophagy, in a cohort of 17 adult ARN patients. With the identification of rare, potentially damaging genetic variants in patients suffering from this rare ocular disease, we hope to shed light on pathways involved in sensing and responding to alphaherpesviruses and to promote understanding of the pathogenesis of viral infection and associated complications in the CNS.

## Materials and methods

### Patient inclusion diagnostic criteria

The study enrolled adult patients from medical Ophthalmology clinics at Rigshospitalet, Copenhagen, University Medical Centre Utrecht, and Halland Hospital Halmstad in the time period from January 2017 until June 2023. The enrolled patients had a clinical diagnosis of acute retinal necrosis in at least one eye fulfilling the criteria described by the American Uveitis Society in 1994 (Holland, 1994). There was no predefined limitation of time interval between the diagnosis of ARN and blood collection for genetic testing. Inclusion criteria involved the presence of necrotizing retinitis affecting the peripheral retina and positive diagnostic tests for either HSV or VZV DNA or antibodies by polymerase chain reaction (PCR) or Goldmann-Witmer Coefficient (GWC) in aqueous or vitreous specimens. In case of the Dutch patients, all individuals have positive results for both tests. The clinical characteristics of all patients meeting the inclusion criteria are summarized in Table 1. Exclusion criteria encompassed the inability to provide informed consent, known primary or secondary immunodeficiency, pregnancy or malignancy, or positive tests for toxoplasmosis and syphilis.

**Table 1:** Demographics and clinical characteristics of the ARN cohort.

Patient ID	Age, Sex	Country	Clinical diagnosis	Medical history	Aqueous humor analysis
P1	70, F	Sweden	ARN secondary to VZV infection	Healthy	PCR
P2	63, F	Sweden	ARN secondary to VZV infection	Healthy (Previously chickenpox as an adult with postinfectious iritis 1989)	PCR
P3	72, F	Sweden	ARN secondary to VZV infection	Pulmonary emboli 2016 and 2019.	PCR
P4	20, F	Denmark	ARN secondary to HSV-2 infection	Autism/Aspergers	PCR
P5	31, M	Denmark	ARN secondary to VZV infection	Healthy	PCR
P6	44, M	Denmark	ARN secondary to VZV infection	Healthy	PCR
P7	28, M	Denmark	ARN secondary to HSV-2 infection	Healthy	GWC
P8	60, M	Denmark	ARN secondary to VZV infection	Healthy	PCR
P9	62, F	Netherlands	ARN secondary to HSV-1	Healthy/hypertension	PCR/GWC
P10	18, F	Netherlands	ARN secondary to HSV-1 infection	Healthy/autism	PCR/GWC
P11	26, M	Netherlands	ARN secondary to VZV infection	Healthy	PCR/GWC
P12	72, F	Netherlands	ARN secondary to VZV infection	Polymyalgia rheumatica (no steroids)	PCR/GWC
P13	24, F	Netherlands	ARN secondary to VZV infection	Healthy	PCR/GWC
P14	58, F	Netherlands	ARN secondary to HSV-1	2015 HSV encephalitis	PCR/GWC
P15	69, F	Netherlands	ARN secondary to VZV infection	Asthmatic bronchitis/hypertension	PCR/GWC
P16	50, M	Netherlands	ARN secondary to VZV infection	Healthy	PCR/GWC
P17	58, M	Netherlands	ARN secondary to VZV infection	Herpes zoster infection 01.22	PCR/GWC

Age stated is the age of each patient upon disease onset. P, patient; ID, identification; f, female; m, male; ARN, acute retinal necrosis; HSV, herpes simplex virus; VZV, varicella zoster virus; PCR, polymerase chain reaction; GWC, Goldmann-Witmer coefficient.

### DNA extraction

Whole blood from P1-P8 was collected aseptically into EDTA containing tubes from each patient. Genomic DNA was isolated from the whole blood with help of the DNeasy Blood and Tissue Kit (Qiagen) according to manufacturer’s instructions. P9-P17 were included at the University Medical Center Utrecht, the Netherlands, genomic DNA purified from blood samples. The samples were selected from the Uveitis Biobank and all patients signed informed consent.

### Whole exome sequencing and bioinformatics

Genomic DNA purified from whole blood samples from P1–P8 was subjected to Whole Exome Sequencing (WES) at the Department of Molecular Medicine (MOMA), Aarhus University Hospital, using KAPA HTP library preparation and Nimblegen SeqCap EZ MedExome Plus kits, followed by analysis with Nextseq version2 chemistry [2 × 150 base pairs] from Illumina. Single nucleotide polymorphisms (SNPs) were called relative to hg19. Variant call files were further filtered using VarSeq 2.3.0 (Golden Helix). Variants were filtered according to (a) quality and read-depth, (b) frequency, only keeping undescribed or rare variants, (c) predicted variant deleteriousness, and (d) biological relevance.

Genomic DNA purified from whole blood samples from P9-P17 was subjected to Whole Genome Sequencing (WGS) at BGI (BGI Europe A/S, Copenhagen, Denmark),^1^ using DNBSEQ™ Sequencing Technology and BGISEQ-500 platform. All clean reads were aligned to the human reference genome (GRCh37/HG19) using Burrows-Wheeler Aligner (BWA V0.7.12). Variant calling was performed using best Practices for variant analysis with the Genome Analysis Toolkit [HaplotypeCaller of GATK(v3.3.0)].^2^ VCF files were annotated using ANNOVAR (Wang et al., 2010).

**Figure 1:**
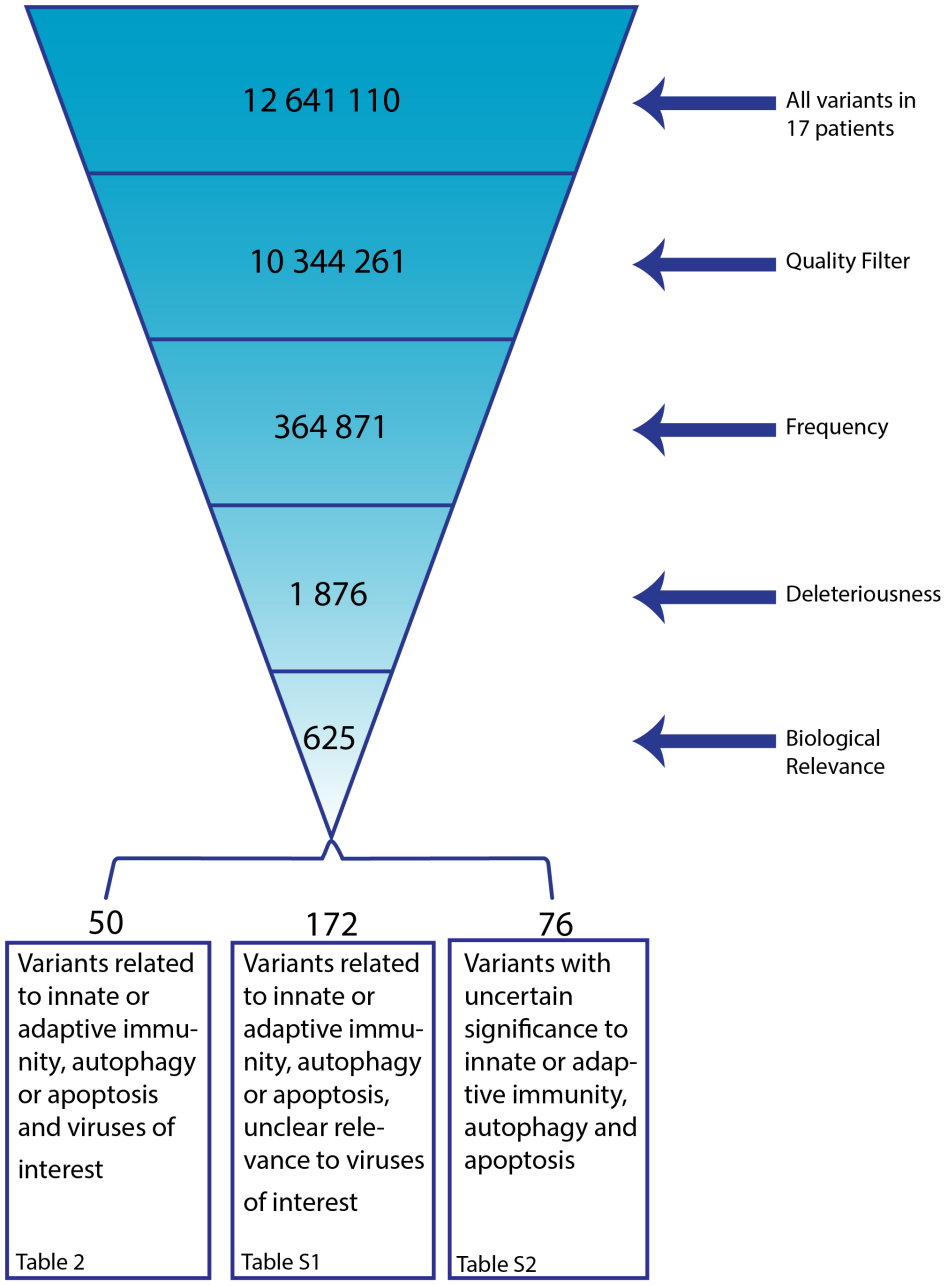
Overview on Filtering Process following Whole Exome Sequencing (WES). From the total of variants identified in 17 patients with alphaherpesvirus-induced ARN we included those of high quality, frequencies of <0.1%, predicted to be Loss of Function (LoF), missense, or splice-site and predicted to be deleterious by at least one out of six damage prediction tools (SIFT, PolyPhen-2, MutationTaster, MutationAssessor, FATHMM, FATHMM MKL). In addition, variants were selected with a CADD score < 15, classified to be Likely Pathogenic, Pathogenic, VUS, VUS/Conflicting, VUS/Weak Pathogenic, VUS/Weak Benign by the ACMG Sample Classifier AND with a REVEL score < 0.25. Variants of biological relevance were subsequently selected. The remaining variants were manually evaluated and listed in Table 2 and Supplementary Tables S1, S2.

Variants were filtered using Tibco Spotfire Analyst according to (a) quality and read-depth, (b) frequency, only keeping undescribed or rare variants, (c) predicted variant deleteriousness and (d) biological relevance. Detailed Description for all Filters (see Figure 1 for overview of filtering of variants):

(a) Quality: only kept variants that pass the Variant Quality Score Recalibration (VQSR) filtering (or missing) AND have a frequency in the sample fitting a hemi-, hetero-, or homozygous phenotype (or missing) AND have a technical read-depth of 30 or more AND achieve a Genotype Quality Score of more than 20
(b) Frequency: exclude variants with reported frequencies >0.1% AND exclude variants in 1% most exonically variable genes in human public database
(c) Deleteriousness: Only keep variants that are LoF, missense OR splice site variants AND only keep variants predicted to be damaging by at least one out of six damage prediction tools [SIFT (Kumar et al., 2009), PolyPhen-2 (Adzhubei et al., 2010), MutationTaster (Schwarz et al., 2014), MutationAssessor (Reva et al., 2011), FATHMM, FATHMM MKL (Shihab et al., 2014)]AND have a CADD score > 15 (Kircher et al., 2014) AND are classified to be Likely Pathogenic, Pathogenic, VUS, VUS/Conflicting, VUS/Weak Pathogenic, VUS/Weak Benign by the ACMG Sample Classifier (Richards et al., 2015) AND have a REVEL score < 0.25 (Ioannidis et al., 2016)
(d) Biological Relevance: using gene lists related to VZV, HSV, immune response, immunodeficiency and autophagy

P9-P17 were included at the University Medical Center Utrecht, the Netherlands, genomic DNA purified from blood samples. Following, the remaining variants were examined manually and sorted in three categories. Variants with roles in innate or adaptive immunity, autophagy or apoptosis and connection to the relevant pathogen are considered to be most likely disease contributing and are displayed in Table 2. Variants in genes with roles in immunity, autophagy or apoptosis but no connection to the virus infection in the patient are collected in Supplementary Table S1. Variants in genes with no connection to the relevant pathogen and non-substantial biological relevance are in Supplementary Table S2. Variants with a MSC score (Itan et al., 2016) higher than CADD score were excluded, as were variants in genes described to be causative for diseases not present in the regarding patient.

**Table 2:** Identified rare genetic variants in patients with ARN.

P ID	Gene Symbol	Biological Category	Transcript ID	Transcript Variant; Protein Variant	Translation Impact	CADD; MSC Scores	SIFT Function Prediction	GnomAD Frequency (%)
**P1**	***ATG9A***	Autophagy	NM_001077198.3	c.823C>T; p.Arg275Cys	Missense	27.7;3.313	Damaging	Not described
**P2**	***TSC2***	Autophagy	NM_000548.5	c.2009>T; p.Pro670Leu	Missense	22.2;0.001	Damaging	0.00007652
	***ASB2***	Innate Immunity	NM_001202429.2	c.403A>C; p.Asn135His	Missense	25.4;3.313	Damaging	0.000007956
**P3**	***SMARCD2***	Innate Immunity	NM_001098426.2	c.1520 T>C; p.Val507Ala	Missense	26.5; 13.187	Damaging	Not described
	***USP5***	Virus Interaction	NM_001098536.2	c.1333A>C; p.Asn445His	Missense	29.3; 5.974	Damaging	0.00008372
	***CRYZ***	Apoptosis	NM_001889.4	c.784A>G; p.Lys262Glu	Missense	24.3; 3.313	Damaging	0.00005185
	***ASB2***	Innate Immunity	NM_001202429.2	c.730G>A; p.Glu244Lys	Missense	27.4; 3.313	Damaging	0.0001592
	***ASB3***	Innate Immunity	NM_016115.5	c.35C>G; p.Ser12Cys	Missense	24.5; N/S	Damaging	0.0002530
	***PLA2G2F***	Adaptive Immunity	NM_022819.4	c.536T>G; p.Val179Gly	Missense	22.4; 3.313	Tolerated	0.0003857
	***AP3B1***	Innate Immunity and Virus Interaction	NM_003664.5	c.604-2A>G	Splice-Site	34.0; 0.879	N/S	Not described
	***THBS1***	Innate Immunity	NM_003246.4	c.885_892delGGACAGCA; p.Gln295Hisfs*6	Frameshift	39.0; 5.805	N/S	Not described
**P4**	***TRIM8***	Innate immunity and autophagy	NM_030912.3	c.1415A>G; p.Asn472Ser	Missense	25.6; 3.313	Damaging	Not described
	***AMFR***	Innate Immunity & Virus Interaction	NM_001144.6	c.1903C>T; p.Arg635Trp	Missense	28.7; 3.313	Damaging	0.000007992
	***OAS2***	Innate Immunity	NM_002535.3	c.1948C>T; p.Arg650Cys	Missense	25.1; 3.313	Tolerated	0.0002547
**P5**	***ATG9B***	Autophagy	NM_001317056.2	c.1748G>A; p.Gly583Asp	Missense	20.7; 3.313	N/S	Not described
**P6**	***CTSB***	Virus Interaction	NM_001908.5	c.835G>A; p.Ala279Thr	Missense	32.0; 3.313	Damaging	Not described
	***TRRAP***	Innate Immunity	NM_003496.4	c.2686G>A; p.Gly896Ser	Missense	26.6; 3.313	Damaging	0.000003978
	***NCSTN***	Innate & Adaptive Immunity	NM_015331.3	c.2108A>T; p.Glu703Val	Missense	27.1; 0.001	Damaging	0.000003977
**P7**	***CDC7***	Virus Interaction	NM_003503.4	c.463C>T; p.Arg155Trp	Missense	29.0; 3.313	Damaging	0.00004799
	***LRRC8A***	Innate & Adaptive Immunity	NM_019594.4	c.385G>A; p.Val129Met	Missense	27.2; 3.313	Damaging	Not described
	***DDX3X***	Innate Immunity	NM_001356.5	c.917T>C; p.Ile306Thr	Missense	24.0; 2.983	Tolerated	Not described
**P8**	***APC2***	Innate & Adaptive Immunity	NM_005883.3	c.638A>G; p.Gln213Arg	Missense	33.0; 3.313	Damaging	Not described
	***APLP2***	Adaptive Immunity & Apoptosis	NM_001142276.2	c.1399C>T; p.Arg467Cys	Missense	29.6; 3.313	Damaging	0.000003978
	***LIF***	Innate Immunity	NM_002309.5	c.475T>C; p.Tyr159His	Missense	25.0; 9.151	Tolerated	0.00001988
	***DCLRE1C***	Adaptive Immunity	NM_001033855.3	c.169G>T; p.Val57Phe	Missense	27.9; 0.531	Damaging	0.0003752
	***TRRAP***	Innate Immunity	NM_003496.4	c.2815A>G; p.Met939Val	Missense	16.4; 3.313	Tolerated	0.00002387
**P9**	***PC***	Innate Immunity	NM_001040716.2	c.2804C>T; p.Ala935Val	Missense	25.1; 9.586	Damaging	0.0002
	***JAK2***	Innate & Adaptive Immunity	NM_004972.4	c.2435-20A>G	Splice-Site	N/S; 23.800	N/S	0.00000401
	***IRF7***	Innate Immunity	NM_001572.5	c.255_262delGCTCCGCA; p.Ala86Argfs*23	Frameshift	N/S; 3.313	N/S	0.00000504
**P10**	***IL17RD***	Innate & Adaptive Immunity	NM_017563.5	c.398C>T; p.Pro133Leu	Missense	24.6; 20.800	Damaging	0.0000888
	***NCBP1***	Virus Interaction	NM_002486.5	c.1084C>T; p.Leu362Phe	Missense	27.5; 3.313	Damaging	Not described
**P11**	***GZMB***	Adaptive Immunity & Virus Interaction	NM_004131.6	c.67G>A; p.Glz23Arg	Missense	26.0; 3.313	Damaging	0.0001
	***IFNGR1***	Innate Immunity	NM_000416.3	c.373+1G>T	Splice-Site	33.0; 0.002	N/S	0.00000398
**P12**	***ITGA6***	Virus Interaction	NM_001079818.3	c.1269+17G>C	Splice-Site	N/S; 22.100	N/S	0.0001
	***DHX36***	Innate Immunity	NM_020865.3	c.343T>G; p.Ser115Ala	Missense	22.6; 3.313	Damaging	Not described
**P14**	***GBP5***	Innate Immunity	NM_052942.5	c.318+20T>C	Splice-Site	N/S; 3.313	N/S	Not described
	***STAT4***	Innate & Adaptive Immunity	NM_003151.4	c.1112+18T>C	Splice-Site	N/S; 3,0.13	N/S	0.0000159
	***CASP10***	Apoptosis	NM_032977.4	c.953G>A; p.Gly318Glu	Missense	23.2; N/S	Damaging	0.0003
	***FLT1***	Innate Immunity	NM_002019.4	c.259_261del; p.Gln87del	In-Frame Deletion	N/S; 3.313	N/S	Not described
**P15**	***ATP2A2***	Autophagy	NM_001681.4	c.1765A>T; p.Asn589Tyr	Missense	26.6; N/S	Damaging	0.00000398
**P16**	***ATG13***	Autophagy	NM_001142673.3	c.1280T>A; p.Leu427Gln	Missense	25.4; 8.153	Tolerated	0.0004
	***MYLK3***	Innate Immunity	NM_182493.3	c.1975C>A; p.Leu659Met	Missense	24.8; 3.313	Damaging	Not described
	***MYT1***	Virus Interaction	NM_004535.3	c.3093+13->C	Splice-Site	N/S; 3.313	N/S	Not described
**P17**	***NCSTN***	Innate & Adaptive Immunity	NM_015331.3	c.967C>T; p.Arg323Cys	Missense	3.,0; N/S	Damaging	0.0000278
	***IRF6***	Innate Immunity	NM_006147.4	c.777G>A; p.Met259Ile	Missense	22.9; N/S	Tolerated	0.0003
	***AMPD3***	Virus Interaction	NM_000480.3	c.1297G>T; p.Val433Phe	Missense	27.8; 13.020	Damaging	0.0000318
	***C3***	Innate Immunity	NM_000064.4	c.193A>C; p.Lys65Gln	Missense	23.6; N/S	Damaging	0.0000477
	***IFIH1***	Innate Immunity	NM_022168.4	c.2417G>A; p.Arg806His	Missense	32.0; 19.330	Damaging	0.0000243
	***ATXN3***	Autophagy	NM_004993.6	c.872_873insCAGCAGCAG CAGCAGCAGCAGCAGCAGC; p.Lys291Asnfs*36	Frameshift	N/S; 3.313	N/S	Not described
	***APC2***	Virus Interaction	NM_005883.3	c.2653C>T; p.Arg885Trp	Missense	23.7; 3.313	Damaging	Not described

Variants identified in patients with ARN. P, patient; ID, identification; ARN, acute retinal necrosis, N/S, not specified; CADD, combined annotation dependent depletion; MSC, mutation significance cutoff; SIFT, Sorting Intolerant from Tolerant; gnomAD, genome aggregation database.

Additionally, variants were filtered using Tibco Spotfire Analyst according to different MOI patterns without filtering for biological relevance:

1. Dominant (patients 0/1, PAV (protein affecting variants), control cohorts (GnomAD, ExAC) <0.0001, CADD (version 1.6) score > 20).
2. Compound heterozygous (control cohorts (GnomAD, ExAC) < 0.03 AND per patient 0/1). Select genes with two different variants per patient.
3. Homozygous (control cohorts (GnomAD, ExAC) < 0.03 AND per patient 1/1).

QIAGEN IPA (QIAGEN Inc.)^3^ was used to get neighborhood genes linked to known candidates (*TLR3, UNC93B1, TRAF3, TICAM1, TBK1, IRF3* and *DBR1*).

Neighborhood genes were added as a column to the variants file in Tibco Spotfire Analyst. Results of this additional filtering can be found in Table 3.

**Table 3:** Selected rare genetic variants in patients with ARN identified through unbiased analysis without biological filters.

P ID	Gene symbol	Transcript ID	Transcript variant; protein variant	Translation impact	CADD; MSC scores	SIFT function prediction	GnomAD frequency (%)
**P8**	***COQ9***	NM_020312.4	c.590T>C; p.Ile197Thr	Missense	24,4	Damaging	0.0001034
**P11**	***COQ8A***	NM_020247.5	c.A1571G p.Gln524Arg	Missense	24,5	Tolerated	0.0000119
	***COQ8A***	NM_020247.5	c.C1821A p.Tyr607X	Stopgain	50	N/S	0.00000795
**P13**	***COQ4***	NM_016035.5	c.C613T p.Arg205X	Stopgain	36	N/S	0.00000795

Variants identified in patients with ARN. P, patient; ID, identification; ARN, acute retinal necrosis, N/S, not specified; CADD, combined annotation dependent depletion; MSC, mutation significance cutoff; SIFT, Sorting Intolerant from Tolerant; gnomAD, genome aggregation database.

### Ethics

Swedish patients P1-3 were included in accordance with The Helsinki Declaration and with approval from the Swedish Ethical board (no. 2020-04850).

Danish patients P4–P8 were included in accordance with The Helsinki Declaration and Danish ethics guidelines after approval from the Danish National Committee on Health Ethics (no. 1-10-72-275-15) and the Data Protection Agency.

Dutch patients P5-P17 were included after approval form The Biobank Research Ethics Committee (TCBio) UMC Utrecht (TCBio-protocolnr. 21-264).

Oral and written consent from each patient was obtained.

### STRING analysis

Variant-containing genes after the last evaluation step (50 genes; Table 2) were subjected to STRING analysis for known and predicted interactions of gene products. Based on (Szklarczyk et al., 2015) Version 11.5, STRING Consortium 2023.

## Results

### Identification of rare genetic variants in patients with alphaherpesvirus induced ARN

For this study, 17 immunocompetent ARN patients from Sweden, Denmark and the Netherlands were included. The patients had clinical signs of ARN and clinical diagnosis was confirmed and viral infection determined by detection of viral DNA and/or intra-ocular production of virus-specific antibodies in aqueous and vitreous humor by real-time quantitative PCR and/or Goldman-Witmer-Coefficient (GWF) (Fekkar et al., 2008). As reported by others, Varicella zoster virus was the most common viral etiology detected in the ARN cohort, namely in 12 patients (70.6%), HSV-1 in three (17.6%) and HSV-2 in two (11.8%). Our cohort thereby follows the distribution previously described and is representative for ARN (Lau et al., 2007; Cox and Miller, 2022). Age of disease onset was between 18 and 72 years, with five patients between 18 and 30 years old (29.4%), six patients between 31 and 60 years (35.3%) and six above 60 years (35.3%). In the youngest group HSV-2 or VZV were identified in two cases each, and HSV-1 in one. In the middle age and oldest group VZV was detected in five cases and HSV-1 for one each. Notably, HSV-2 only occurred in the youngest age group in our cohort, which is in concordance with this virus being described as more common among young ARN patients (Cox and Miller, 2022). Only P14 suffered from a previous episode of encephalitis, which is described as risk factor for ARN (Ahmadieh et al., 1991; Levinson et al., 1999; Ganatra et al., 2000; Maertzdorf et al., 2001; von Hofsten et al., 2019). More detailed demographic and clinical information of the ARN patients is provided in Table 1.

Despite the advanced age of some of the patients, the development of ARN by common human alphaherpesviruses reactivating from latency is a rare event, given the rarity of the disease suggesting an underlying IEI. To identify potential genetic causes for the infectious phenotype we subjected DNA from all patients to WES analysis and analyzed genetic variants relative the human reference genome (GRCh37/HG19). Subsequently, these identified variants were filtered by quality and read-depth, frequency predicted variant deleteriousness and biological relevance. Figure 1 shows a schematic of the filtering process. In total, 625 variants were left after automatic filtering. Of these, 327 variants were excluded based on manual quality control, if the combined annotation dependent (CADD) score < mutation significance cut-off (MSC), if present in a low complexity region of the genes that is known to be difficult to sequence or if the gene was described as causative for a disease the regarding patient did not have and that would have developed by age of ARN diagnosis. The remaining 298 variants were sorted in three categories. First, 50 variants in genes involved in immune signaling, autophagy or apoptosis, having been described to play a role in infection with the associated virus. These have the highest probability of being disease contributing/causing (Table 2). Second, 172 variants were found in genes with a role in immune signaling, autophagy or apoptosis, but without any known direct involvement in VZV or HSV antiviral pathways (Supplementary Table S1). Lastly, 76 variants in genes without evidence of a substantial role in immune signaling, autophagy, apoptosis or virus interaction remained (Supplementary Table S2). In 16 of 17 patients, the search for potentially disease contributing variants was successful (Table 2). The number of variants per patient ranges from one to eight. Among the 50 variants presented in Table 2, 39 are missense, three are frameshift, seven splice site variants, and one an in-frame deletion.

Remarkably, despite the small size of the cohort, variants in a specific gene occurred in more than one patient. For instance, P2 and P3 display unique variants in *ASB2*. Additionally, P3 harbors a variant in *ASB3* from the same gene family. This accumulation of variants might indicate a prominent role for ASB proteins in preventing ARN. The same holds true for *TRRAP* with variants in P6 and P8, *NCSTN* with variants in P6 and P17 and *APC2* with variants in P8 and P17. Similarly, P1 has a variant in *ATG9A* and P5 in its paralog *ATG9B*, suggesting a possible important role for these genes within the autophagy pathway.

### Variants in innate and adaptive signaling pathways

**Figure 2:**
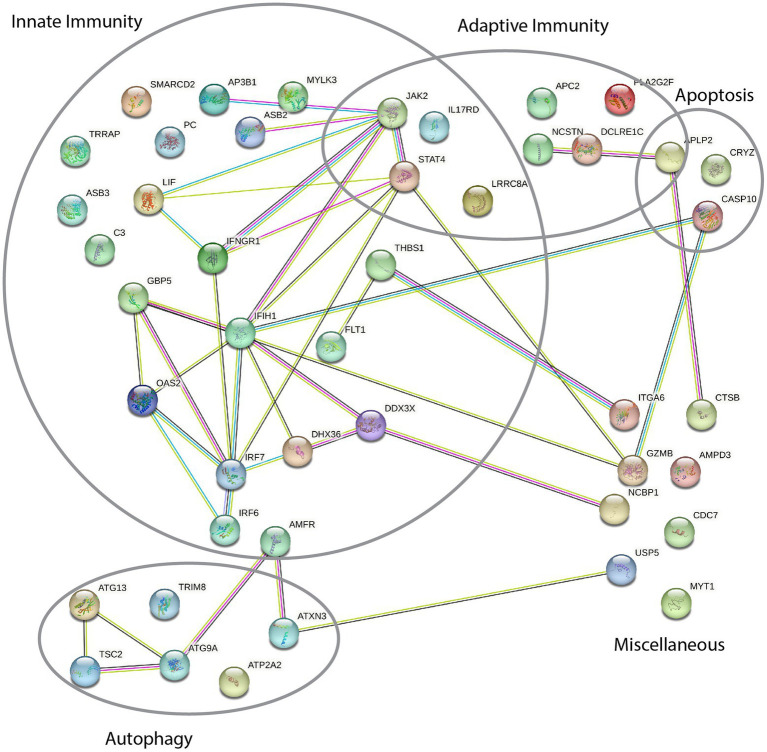
STRING protein–protein interaction network of proteins implicated in the acute retinal necrosis (ARN) patient cohort. Each node depicted represents the gene-product of genes with variants in at least one patient in our ARN cohort. Each line indicates known or predicted interactions between the proteins: Light blue, known interaction from curated database; magenta, known interaction experimentally determined; dark green, predicted interaction based on gene neighborhood; red, predicted interaction based on gene fusion; dark blue, predicted interaction based on gene co-occurrence; light green, text mining; brown co-expression, purple protein homology.

Among the identified variant containing genes, similar functions and common signaling pathways are shared, strengthening the hypothesis of an underlying functional defect. We used STRING analysis to check for protein–protein interaction within the 45 genes from Table 2. The analysis reveals five partly overlapping main clusters of different gene functions based on known and predicted protein interactions (Figure 2). Further, the analysis of the identified genes shows significantly more interactions than expected from a random group of 45 genes (39 interactions instead of 18, protein–protein interaction enrichment value of *p* = 1.02 × 105). This enrichment further indicates functional biological connectivity and involvement of dysfunction of the indicated pathways in the development of ARN.

The largest group of variants lies within genes with a function in innate immunity. Here, a number of genes directly involved in the IFN response were identified, including *IRF7* (P9), *IFNGR1* (P11) and *IRF6* (P17). Analogously, *DDX3X* (P7) leads to IFN-β induction mediated by IRF3 and TBK1 and IKBKE. Also, *IFIH1* (MDA5; P17) senses viral nucleic acids directly in the cytoplasm, leading to induction of type I IFN and IFN-induced genes (ISG) (Gu et al., 2013; Lamborn et al., 2017; Wu et al., 2020; Liu et al., 2021). Importantly, transcription of IFIH1 has been shown to be highly elevated in VZV-infected cells (Oh et al., 2019). *OAS2* (P4) is an IFN-inducible gene activated by dsRNA and regulating antiviral activity *via* RNAseL, which has been ascribed a role in the context of HSV-2 infection (Sarkar et al., 1999a,b; Nixon et al., 2014). Two of the variants are in genes involved in sensing of dsRNA, *PC* (P9) and *DHX36* (P12). PC acts as an activator of the dsRNA sensor RIG-I (Vastag et al., 2011), while DHX36 is a part of a TICAM1 complex, which also senses dsRNA (Kim et al., 2010). Production of significant amounts of dsRNA is a common feature in viruses with a DNA genome (Weber et al., 2006). Several genes are also involved in the JAK–STAT signaling pathway induced by type I IFN, a pathway known to be counteracted by herpesviruses (Yokota et al., 2004; Gerada et al., 2020). Among these are *LiF* (P8), *STAT4* (P14), *ASB2* (P2 & P3) and *ASB3* (P3), and importantly *JAK2* (P9). STAT4 also plays a role in adaptive immunity as a key factor for differentiation of T-helper cells (Morinobu et al., 2002; Zhang et al., 2022).

Within innate immunity, several other well represented pathways among variants are the family of MAPK and, more specifically, JNK signaling. Notably, these pathways have been demonstrated to be upregulated in VZV infection, highlighting their relevance in this context (Hargett et al., 2005; Kurapati et al., 2017; Hu et al., 2020). As an example, *TRRAP*, mutated in P6 and P8, interacts with ELK1, a substrate of JNKs (Zeke et al., 2016). Interestingly, replication efficiency of VZV has been shown to be increased upon inhibition of the JNK pathway (Rahaus et al., 2004). Similarly, IL17RD (P10) also regulates JNK signaling and TLR signaling (Girondel and Meloche, 2021). Additionally the IL17 receptor has been ascribed a role in HSV-1 infection (Hirose et al., 2020). FLT1 (P14) activates the MAPK signaling pathway and its expression is increased in HSV-infected sensory neurons (Kramer et al., 2003). The Wnt/β-catenin signaling circuit is another pathway known to play a role in innate and adaptive immunity and to be manipulated by herpesviruses (Zwezdaryk et al., 2016; Ljungberg et al., 2019). Variants were identified in Wnt signaling regulators *APC2* (P8 & P17), *NCSTN* (P6 & P17) and *AMFR* (P4). AMFR further acts as a ubiquitin-protein ligase (Shimizu et al., 1999).

Moreover, variants were found in *SMARCD2* (P3), involved in differentiation of neutrophils (Witzel et al., 2017), *THBS1* (P3), involved in regulation of dendritic cell activation (Doyen et al., 2003), the activator of inflammasome assembly *GPB5* (P14) (Shenoy et al., 2012), the complement factor C3 (C17), *MYLK3* (P16), involved in IL1β and NF-κB signaling, *AP3B1* (P3), affecting natural killer (NK) and NKT cell granule-associated protein release (Shin J. et al., 2021) and *TRIM8* (P4). TRIM8 has broad roles in innate immunity within IFN-γ, TNF-α and IL1β signaling (Toniato et al., 2002; Yan et al., 2017; Ye et al., 2017). Interestingly, TRIM8 also regulates immune responses by mediating autophagic degradation of cytoplasmic regulators of innate immunity, thus acting at the interface between innate immunity and autophagy in human cells (Kimura et al., 2015). Similarly, *LRRC8A* (P7) has a role as 2′-3’-cGAMP transporter and is involved in B-cell development, bridging between innate and adaptive immunity. Finally, *PLA2G2F* (P3) and *DCLRE1C* (P8) only have roles in adaptive immunity, within V(D)J recombination (Ma et al., 2002; Murakami et al., 2018). PLA2G2F expression is altered in context of VZV infection (Wu et al., 2021). Deficiency of the *DCLRE1C* gene product ARTEMIS leads to severe combined immunodeficiency deficiency, while partially impaired function has been described with persistent VZV infection (Volk et al., 2015). VZV is the ARN causing pathogen in both P3 and P8.

### Variants in the autophagy complex

Autophagy is another main functional group with identified variants in the ARN cohort. As mentioned above, we identified variants both in *ATG9A* (P1) and *ATG9B* (P5), phospholipid scramblases with key roles in autophagosomal membrane expansion (Orsi et al., 2012). *ATP2A* (P15) is involved in formation of autophagic membranes as well (Zhao et al., 2017). Furthermore, we present variants in *ATG13* (P16), part of the autophagy initiation complex (Jung et al., 2009), as well as in *TSC2* (P2) and *ATXN3* (P17) playing roles in initiating autophagy (Ashkenazi et al., 2017; Rehbein et al., 2021). Some of these, like ATG13 and ATXN3, are upregulated in VZV-infected cells (Grose et al., 2016), whereas, e.g., ATP2A2 is downregulated upon infection (Jones and Arvin, 2003). Upregulation of these autophagy proteins upon infection may suggest a role in anti-viral defense whereas downregulation indicates their expression being counteracted by the virus, both leading to the theory that abolished gene function might be deleterious for a functional antiviral immune response. All individuals in which we identified variants in the above mentioned genes are VZV ARN patients.

### Variants in apoptosis pathways

Three of the identified variant containing genes encode proteins with a main role in apoptosis: *CRYZ* (P3) stabilizes BCL2, with anti-apoptotic function in VZV infected cells (Kennedy et al., 2020) and *APLP2* (P8) interferes with apoptosis. Importantly, APLP2 also reduces MHC class I expression, providing it with a double role in apoptosis and adaptive immunity (Pandey et al., 2016). Meanwhile, *CASP10* (P14) activates the apoptotic cascade (Wang et al., 2001). Interestingly, variants in genes with anti-apoptotic function are found in patients with ARN secondary to VZV infection, whereas the variant in a pro-apoptotic gene is found in an HSV ARN patient, possibly indicating different functions of apoptosis in VZV versus HSV infection.

### Variants in miscellaneous signaling pathways

Several other cellular and immunological pathways and molecules were implicated through the presence of gene variants in this analysis. A variant was identified in the gene encoding for the deubiquitinating enzyme USP5 (P3), that modulates neuropathic and inflammatory pain by interacting with calcium channels also implicated in postherpetic neuralgia (García-Caballero et al., 2014; Li et al., 2021). *CTSB* (P6) is a lysosomal protease that is upregulated in VZV infected fibroblasts (Tommasi et al., 2020), and *GZMB* (P11), a cytotoxic molecule secreted by cytotoxic T lymphocytes and NK cells to induce apoptosis and able to cleave viral proteins, combining several different pathways (Tibbs and Cao, 2022). Importantly, among GZMB’s target viruses are both HSV and VZV (Gerada et al., 2019). ITGA6 (P12) is a cell surface receptor downregulated by VZV (Bowles et al., 2012). MYT1 (P16) phosphorylates CDC2, which in turn phosphorylates VZV glycoprotein I. AMPD3 (P17) is a metabolic enzyme alternatively expressed in MeWo-IE63 cells compared with control cells (Habran et al., 2007), indicating possible relevance in context of VZV infection. The Patients 3, 6, 11, 12, 16 and 17 were diagnosed with ARN secondary to VZV infection. *CDC7* (P7) is a kinase involved in the cell division cycle and HSV-1 infection (Hou et al., 2017). Lastly, *NCBP1* (P10) is involved in splicing and translation regulation and decay and nuclear mRNA export out with particular importance in cellular stress situations, such as viral infection (Gebhardt et al., 2015). Furthermore, NCBP1 is differentially expressed after HSV infection (Dembowski and DeLuca, 2015). P7 and P10 developed ARN secondary to HSV-2 and HSV-1, respectively.

### Additional unbiased analysis without biological filters

Finally, a different filtering approach was added. In short, pre-determined gene lists for filtering of biological relevance were left out while other quality and damage prediction filters were even more strict (more details described in the method section). The main findings of this filtering method are variants in different subunits of Coenzyme Q which are shown in Table 3. A variant in *COQ4* was found in P13, and additional variants in *COQ9* and *COQ8A* were identified in P8 and P11, respectively. All variants are of very low frequency and were not present in a cohort of 1,354 healthy individuals above the age of 80 years used as a control cohort (Erikson et al., 2016). Coenzyme Q is an essential component of the mitochondrial respiratory chain, localized to the inner mitochondrial membrane and linked to mitochondrial diseases in general (Desbats et al., 2015). Its dual role as key electron transfer factor in the mitochondria, and as the main antioxidant in the prevention of oxidative damage in cell membranes makes it an essential molecule in cellular survival. LoF mutations in COQ genes have been previously described causative for primary COQ10 deficiency (Blumkin et al., 2014; Desbats et al., 2015; Traschütz et al., 2020). Neurological abnormalities that can occur in primary coenzyme Q10 deficiency include seizures, intellectual disability, poor muscle tone (hypotonia), involuntary muscle contractions (dystonia), progressive muscle stiffness (spasticity), abnormal eye movements (nystagmus), and of note, vision loss caused by retinal degeneration (Blumkin et al., 2014; Desbats et al., 2015; Traschütz et al., 2020). While not traditionally regarded as part of the immune system, *COQ8A* is present in the list of neighborhood genes for TLR signaling regulator *UNC93B1* (Hubel et al., 2019) and preclinical and clinical studies have shown that COQ10 has anti-inflammatory and antioxidant effects, as well as effects on mitochondrial function, which have been linked to the inflammatory response (Sifuentes-Franco et al., 2022), thereby opening up the possibility of a functional role of *COQ10* gene variants in ARN pathogenesis.

## Discussion

In this study, we present the WES analysis of a cohort consisting of 17 ARN patients secondary to VZV (*n* = 12), HSV-1 (*n* = 3) and HSV-2 (*n* = 2) infection. Thereby, we are aiming to unravel presently unknown host-virus interactions and restriction factors and signaling pathways essential for antiviral defenses against herpesviruses in the ocular tissue and the CNS. Remarkably, in 16 out of 17 patients we were able to identify rare genetic variants predicted to be damaging. In total, we here describe 50 rare variants in 45 separate genes predicted to be deleterious in gene function by different bioinformatic damage prediction tools. The gene products have functions within viral sensing, innate and adaptive immunity, autophagy or apoptosis and have previously been described in context of alphaherpesvirus infection. This strict selection process ensures that the described variants are prime candidates for causing infectious susceptibility and pathology, thereby representing possible novel candidates for IEI in humans.

STRING analysis of all variants shows significantly more interactions than expected for a random group of genes (39 instead of 18, Figure 2). This can partly be explained by the use of pre-selected gene lists during the analysis, but nevertheless indicates an enrichment of related pathways in variants of the cohort. Further, the analysis indicates that most variant containing genes can be classified into one or more biologically functional groups: innate immunity, adaptive immunity, autophagy and apoptosis.

### Major findings within innate and adaptive immunity

The innate immune response plays a critical role in herpesvirus pathogenesis (Moraru et al., 2012; Caselli et al., 2017; Gerada et al., 2020); it is therefore not surprising that many IEI previously described by us and others are located within the innate immune system (Roesler et al., 1999; Dupuis et al., 2003; Zhang et al., 2007; Andersen et al., 2015; Kreins et al., 2015; Thomsen et al., 2021). In the present study, the largest functional group of variants lies within innate immune response genes (see Figure 2), indicating functional impairment of innate immune sensing and signaling as one of the leading causes of severe disease in ARN patients. With *IRF7*, *IFNGR1*, *IRF6, DDX3X, IFIH1*, and *OAS2* we identified several variants in genes directly involved in the IFN response, implicating that diminished function of any of these could impair the IFN response upon viral reactivation, potentially facilitating viral spread to the CNS and the ocular nerve. As stated in the introduction, single gene IEIs in genes involved in the IFN response have been found to be causative for both HSV and VZV CNS infection. For example, *DDX3X* mutations in patients with Natural killer/T-cell lymphoma have been found to underly disturbances of NK cell function (Jiang et al., 2015), ASB2 is involved in NK cell migration (Shin J. H. et al., 2021) and Lif has been described to alter receptor expression on NK cells (Abdoli et al., 2021). In addition, STAT4 appears to be imperative for IL-12 mediated cytotoxicity and IFN-γ production in NK cells (Wang et al., 2000). NK cell function is found to be of particular importance in herpesvirus virulence (Moraru et al., 2012; Caselli et al., 2017), and NK deficiency was among the first described predisposing factors for severe VZV infection (Biron et al., 1989; Gineau et al., 2012).

Most remarkably, despite the relatively small size of the cohort and the rareness of the disease in general, we found not only variants in related pathways, but in the same gene in different patients. This serves as a strong indicator for the importance of proper gene function for prevention of ARN in VZV/HSV infection. *ASB2* and *ASB3* with variants identified in several patients, play a role in suppression and degradation of cytokines, such as JAK2 (Kohroki et al., 2005; Nie et al., 2011). Given that ARN is characterized by excessive inflammation in the eye, it is possible that uncontrolled activation of JAK–STAT signaling will lead to pathological inflammation, thereby contributing to the observed necrosis. ASB2 is strongly upregulated in VZV-infected cells (Kim et al., 2015). Similar considerations may be made for the variants present in the histone acetyltransferase *TRRAP*, a protein that alters gene transcription in response to binding with phosphorylated Elk1, a JNK substrate (Zeke et al., 2016). TRRAP plays a role in repressing IFN stimulated genes, pointing toward excessive inflammation in the absence of impaired TRRAP function (Detilleux et al., 2022).

### Variants involved in sensing of viral RNA in herpesvirus infection

Intriguingly, two of the identified variants, *DHX36* and *PC*, are within genes related to sensing of viral RNA, in accordance with a failure to detect dsRNA intermediates of both viruses would be detrimental to the host. Previously, a number of patients with severe VZV infection were demonstrated to harbor gene variants in different subunits of RNA polymerase III, a cytosolic DNA sensor converting AT-rich DNA into 5′ 3-phosphorylated RNA (Ogunjimi et al., 2017; Carter-Timofte et al., 2018a,b, 2019; Thomsen et al., 2021). Therefore it may appear somewhat surprising that in this cohort with 12 patients developing ARN secondary to VZV infection we did not observe any potentially disease causing variants in POL III genes under the defined selection criteria for deleteriousness and frequency. Among the previously identified patients with defective POL III sensing were children with encephalitis, cerebellitis and pneumonia as well as adults diagnosed with meningoencephalitis and CNS vasculitis (Ogunjimi et al., 2017; Carter-Timofte et al., 2018a, 2019). Our main explanation for why POL III variants were not identified in the present ARN cohort is either the cohort is relatively small and therefore not given to identify POL III variants which are only present in a minority of patients with VZV infection, or that ARN is mainly a reactivation event, whereas most, but not all, POL III variants were originally identified in children with VZV encephalitis during primary infection (varicella) – immune mechanism may differ and involve different molecules and pathways in primary infection as opposed to reactivation in the eye to cause ARN. Intriguingly, however, two of the identified variants, *DHX36* and *PC*, are within genes related to sensing of viral RNA, in accordance with a failure to detect dsRNA intermediates of both viruses would be detrimental to the host.

### Defects in autophagy pathways and processes

A particular interesting finding of the present study was several variants in autophagy-related genes. *ATG9A* and its paralogue *ATG9B* encode autophagy proteins with are key roles in autophagic membrane elongation (Orsi et al., 2012). Additional variants in autophagy related genes were present within this cohort, mostly involving initiation of autophagy. For a long time, autophagy has been known as an anti-viral defense mechanism (Lee and Iwasaki, 2008). With regards to HSV-1 infection, there is good evidence that autophagy plays a protective role, especially in the CNS (Wilcox et al., 2015; Yakoub and Shukla, 2015). We have previously described two patients with variants in autophagy-related genes contributing to development of recurrent HSV2 lymphocytic Mollaret meningitis (Hait et al., 2020) and have described more patients harboring autophagy related gene variants (Hait et al., 2021). Whereas it has been demonstrated that autophagy is upregulated in VZV infection (Eisfeld et al., 2006; Takahashi et al., 2009; Girsch et al., 2020); the importance of autophagy in antiviral immunity against VZV is more controversial and unresolved. (Extensively reviewed in Heinz et al., 2021).

Previous work from our group on a VZV encephalitis cohort also showed a significant portion of variants within the autophagy pathway (Thomsen et al., 2021), implicating variants in *ATG2A* and *ATG2B, ULK1, TGM2* and *LAMP2*, alongside yet another variant in *ATG9A* and one in *TSC1*. Accumulation of variants in autophagy-related genes in VZV CNS infection (Thomsen et al., 2021) and the present ARN cohort may suggest autophagy defects to be unfavorable for these patients. For this reason, we believe the description of novel variants in relation to VZV infection provides especially valuable information about the relevance of this pathway in VZV antiviral defenses.

### A role for dysregulated apoptosis in ARN development

Genetic variants in apoptosis-related genes were previously identified in poliomyelitis and a Mollaret meningitis cohort (Andersen et al., 2019; Hait et al., 2021); however, the present study represents the first description of major variants related to apoptosis in patients with VZV infection. Considering how herpesviruses establish latency in their hosts it seems plausible that a careful regulation of cell death pathways is of central importance. Here we identified two variants in anti-apoptotic genes, the impaired functionality of which might lead to excessive cell death thereby possibly contributing to necrosis of ocular tissue. On the other hand, we report one variant in a pro-apoptotic gene, indicating that impaired apoptosis might interfere with viral control earlier in the re-activation phase and thereby enable increased production of viral particles, which in turn increases the risk for viral dissemination to the ocular nerve. Functional studies are needed to further elucidate this fascinating potential role for apoptosis dysregulation in severe infection.

### Variants in other signal transduction pathways

The canonical Wnt signaling pathway, of which NCSTN belongs, is established to provide anti-viral activity against different herpesviruses (Zwezdaryk et al., 2016). Intriguingly, Wnt signaling is described to be upregulated in VZV infected neurons, but not in fibroblasts (Markus et al., 2014), indicating a particular importance of this pathway in the context of CNS infection. Moreover, not only Wnt signaling, but also NCSTN is significantly upregulated in VZV infection (Jones and Arvin, 2003). However, the exact mechanism by which reduced function of NCSTN may benefit VZV needs further experimental validation.

### Coenzyme Q10 deficiency in ARN

Finally, we found potentially damaging variants in *COQ4*, *COQ8A* and *COQ9* in three unrelated patients suffering from VZV-induced ARN. The co-enzyme Q10 complex plays a role in inhibition of ROS production by reducing oxidative damage and inflammation and; importantly, is expressed in the retina (Qu et al., 2009). A normal functioning COQ10 complex reduces cell death of retinal cells and delays progression of retinal diseases such as age-related macular degeneration, retinitis pigmentosa, diabetic retinopathy, glaucoma and last but not least ARN (Zhang et al., 2017; Verbakel et al., 2018; Jurkute et al., 2022; Fuller et al., 2023). Of note, VZV, the diagnosed pathogen in all three patients with COQ variants, is known to disrupt proper mitochondrial function and production of reactive oxygen species (Keller et al., 2016; Andrieux et al., 2021). Evolutionary conservation of this pathway has been described in *Drosophila melanogaster* (Hura et al., 2022), further underlining the essential role of this biological pathway, possibly also in antiviral defense against VZV infection of ocular tissue. Of note, Q10-related variants have not previously been identified in any other WES studies (Andersen et al., 2019; Hait et al., 2021; Thomsen et al., 2021), underlining a potential specificity of COQ10 biology for ARN development.

### Limitations of the study

The present analysis represents the largest cohort of ARN patients investigated genetically, particularly given the rareness of the disease. Our findings can be seen as a starting point for furter studies on the pathogenesis of ARN, a knowledge that will in the long term be of advantage for prognosis, prevention and treatment methods. However, confirmation of these findings in larger cohorts is needed. Perhaps most importantly, a tremendous amount of work is left to functionally evaluate the importance of each of the identified genes as well as the mechanism by which defective function of that gene promotes development of ARN. Each of the variants must be functionally validated through knock-out and Knock-in approaches in patient cells and experimental cell models, and the patient cells must be reconstituted to normal by overexpression of the wild-type protein of interest. The incompleteness of the method represents another limitation of the study: since WES analysis does not cover intronic and non-coding regions of the genome some disease causing genetic alterations may be missed. Although most disease causing variants presently known are localized within the coding regions, there is a risk of missing potentially disease causing variants in introns and regulatory regions of the genome. Another interesting, so far unanswered, question is, whether the identified gene defects lead to more frequent viral reactivation, or do not change the frequency of reactivation *per se*, but rather increase the risk of developing complications in response to these. Indeed, genetic defects leading to more frequent reactivation have been described previously (Estefanía et al., 2007; Rizzo et al., 2012; Caselli et al., 2014), although for other IEI underlying severe viral disease, the pathogenesis rather involves unrestricted replication once the virus is reactivated.

## Conclusion

Here, we identify several genes and pathways that appear to be important for disease pathogenesis. Further functional studies on each of these gene variants are necessary in the future. While in some cases disease phenotype will not be explained by a single-gene IEI, overall, we believe that our findings contribute to explaining the development of severe retinal disease in at least some of these patients and provide new insight into the pathomechanisms of severe alphaherpesvirus infection of the brain and related tissues. Collectively the present manuscript represents a descriptive genetic study with the aim of providing a map of some genes/molecular targets and pathways in immune circuits and restriction factors within cells that may be disturbed or defective in patients with insufficient immune responses or inability to maintain latency of VZV and HSV1 and 2. These results may then inspire further experimental studies and comparison of identified variants by whole exome/whole genome sequencing in patients with VZV, HSV1 or similar severe viral infections. Ultimately, we expect this knowledge to be valuable for future development of improved patient diagnosis, prophylaxis, and treatment of this rare, yet sight-threatening ocular disease and to elucidate so far unknown aspects of neuropathogenesis of alphaherpesvirus infection in humans.

## Data availability statement

The datasets presented in this article are not readily available because Danish GDPR rules do not allow sharing entire genetic sequences from humans in a repository. Requests to access the datasets should be directed to TM.

## Ethics statement

The studies involving humans were approved by Swedish patients P1-3 were included in accordance with The Helsinki Declaration and with approval from the Swedish Ethical board (no. 2020-04850). Danish patients P4–P8 were included in accordance with The Helsinki Declaration and Danish ethics guidelines after approval from the Danish National Committee on Health Ethics (no. 1-10-72-275-15) and the Data Protection Agency. Dutch patients P5-P17 were included after approval form The Biobank Research Ethics Committee (TCBio) UMC Utrecht (TCBio-protocolnr. 21-264). Oral and written consent from each patient was obtained. The studies were conducted in accordance with the local legislation and institutional requirements. The participants provided their written informed consent to participate in this study.

## Author contributions

TM and GV conceived the idea. TM, JHo, TI, MH, and JB cared for patients. JHe, MT, JHo, TI, MH, and JB collected and isolated patient material. JHe analyzed data together with MT, SS, PS, PH, KK, and TM. TM and JHe wrote the first draft of the manuscript with contributions from GV, JHo, TI, MH, JB, MT, KK, SS, PS, and PH. TM and JHe revised the manuscript. All authors contributed, read, commented, and approved the final version of the manuscript.

## Figures and Tables

**Table:** 

ARN	acute retinal necrosis
CNS	central nervous system
ds	double stranded
ELISA	enzyme-linked immunosorbent assay
GWC	Goldmann-Witmer coefficient
HSV	herpes simplex Virus
IEI	inborn errors of immunity
IFN	interferon
LoF	loss of function
NK	natural killer
PCR	polymerase chain reaction
PID	primary immunodeficiency
SCID	severe combined immunodeficiency
WES	whole exome sequencing
VZV	varicella-zoster virus

## Footnotes

^1^www.bgisequence.com

^2^https://www.broadinstitute.org/gatk/guide/best-practices

^3^https://digitalinsights.qiagen.com/IPA
