# Supplementary material for: Whole exome sequencing of patients with varicella-zoster virus and herpes simplex virus induced acute retinal necrosis reveals rare disease-associated genetic variants
Source: Front Mol Neurosci. 2023 Oct 25;16:1253040. doi: 10.3389/fnmol.2023.1253040 (PMC10630912; doi:10.3389/fnmol.2023.1253040)
Supplement: Supplementary file 1 [file Table_1.DOCX]

## **Supplementary Table 1.** **Variants in genes related to immune signaling, autophagy or apoptosis excluded based on uncertain relevance to VZV/HSV infection**

| P ID | Gene Symbol | Biological Category | Transcript ID | Transcript Variant; Protein Variant | Translation Impact | CADD; MSC Scores | SIFT Function Prediction | GnomAD  Frequency  (%) |
| --- | --- | --- | --- | --- | --- | --- | --- | --- |
| P1 | *NRP1* | Innate Immunity & Virus Interaction | NM_003873.7 | c.1699G>A;  p.Glu567Lys | Missense | 31.0; 3.313 | Damaging | 0.000003977 |
|  | *MSN* | Adaptive immunity | NM_002444.3 | c.1123C>G;  p.Leu375Val | Missense | 23.9; 16.612 | Damaging | Not described |
|  | *GLA* | Innate Immunity | NM_000169.3 | c.427G>A  p.Ala143Thr | Missense | 24.9; 0.085 | Damaging | 0.0005062 |
|  | *DDRGK1* | Innate Immunity & Autophagy | NM_023935.3 | c.605G>A;  p.Gly202Asp | Missense | 26.5; 8.323 | Damaging | 0.00005662 |
|  | *ADGRE2* | Innate Immunity | NM_013447.4 | c.2231C>T;  p.Thr744Met | Missense | 23.5;  N/S | Damaging | 0.00006014 |
|  | *MGST1* | Innate Immunity | NM_020300.5 | c.217C>T;  p.Arg73Cys | Missense | 28.3; 6.517 | Damaging | 0.0001753 |
|  | *PTGS1* | Innate Immunity | NM_000962.4 | c.826C>T;  p.Arg276Ter | Stop Gain | 34.0; 3.313 | N/S | 0.00001062 |
|  | *VIM* | Virus Interaction | NM_003380.5 | c.690G>C;  p.Glu230Asp | Missense | 25.0; 6.123 | Damaging | Not described |
|  | *SPNS2* | Innate Immunity | NM_001124758.3 | c.1607+6T>G | Splice-Site | 22.7; 7.488 | N/S | Not described |
|  | *SPNS2* | Innate Immunity | NM_001124758.3 | c.1607+5_1607+6insGG | Splice-Site | 25.4; 7.488 | N/S | Not described |
|  | *CPNE3* | Autophagy | NM_003909.5 | c.958_960delAAT;  p.Asn320del | In-Frame Deletion | 21.5; 5.764 | N/S | 0.0005339 |
|  | CCDC177 | Virus Interaction | NM_001271507.2 | c.133T>G;  p.Ser45Ala | Missense | 17.6; 3.313 | N/S | Not described |
| P2 | *CFH* | Innate Immunity | NM_000186.4 | c.350G>C;  p.Gly117Ala | Missense | 33.0; 0.001 | Damaging | Not described |
|  | *SPHK2* | Innate Immunity | NM_020126.5 | c.457T>G;  p.Trp153Gly | Missense | 26.2; 3.313 | Damaging | Not described |
|  | *ZFHX3* | Innate Immunity | NM_006885.4 | c.3257G>A;  p.Cys1086Tyr | Missense | 26.2; 3.313 | Damaging | Not described |
|  | *SNX30* | Autophagy | NM_001012994.2 | c.425A>C;  p.Lys142Thr | Missense | 27.6; 3.313 | Damaging | 0.00005339 |
|  | *FBXO30* | Innate & Adaptive Immunity | NM_032145.5 | c.337G>T;  p.Asp113Tyr | Missense | 27.1; 3.313 | Damaging | 0.00001592 |
|  | *TRPM1* | Autophagy | NM_002420.6 | c.1349G>A;  p.Arg450Gln | Missense | 22.6; 0.091 | Damaging | 0.00005745 |
|  | *APPL2* | Innate Immunity | NM_018171.5 | c.992T>C;  p.Met331Thr | Missense | 23.9; 3.313 | Tolerated | 0.0007990 |
|  | *NOD1* | Innate Immunity | NM_006092.4 | c.434T>C;  p.Leu145Pro | Missense | 21.0; 3.313 | Tolerated | 0.0001888 |
| P3 | *SPHK2* | Innate Immunity | NM_020126.5 | c.457T>G;  p.Trp153Gly | Missense | 26,2; 3,313 | Damaging | Not described |
|  | *HAND2* | Innate Immunity & Autophagy | NM_021973.3 | c.275T>G;  p.Leu92Arg | Missense | 22.3;  3.313 | Damaging | 0.00004871 |
|  | *CREBBP* | Innate Immunity | NM_004380.3 | c.2318C>A;  p.Pro773Gln | Missense | 17.1;  0.280 | Tolerated | Not described |
|  | *BRWD1* | Adaptive Immunity | NM_033656.4 | c.3900+3A>T | Splice-Site | 24,3;  3,313 | N/S | 0.00003487 |
|  | *TNKS2* | Innate Immunity | NM_025235.4 | c.570delA;  p.Glu191Lysfs*3 | Frameshift | 32.0;  11.999 | N/S | Not described |
|  | *GBP2* | Virus Interaction | NM_004120.5 | c.451C>T;  p.Arg151Ter | Stop Gain | 33.0;  3.313 | Damaging | 0.00002399 |
|  | HOXD11 | Autophagy | NM_021192.3 | c.612_613insCCCG; p.Glu205Profs*85 | Frameshift | 36.0;  7.290 | N/S | Not described |
|  | HOXD11 | Autophagy | NM_021192.3 | c.608delA; p.Gln203Argfs*89 | Frameshift | 20.7;  7.290 | N/S | Not described |
| P4 | *CYB5R3* | Virus Interaction | NM_000398.7 | c.757G>A;  p.Val253Met | Missense | 26.3;  3.415 | Damaging | 0.0001031 |
|  | *MADD* | Innate Immunity | NM_003682.4 | c.4633G>A;  p.Glu1545Lys | Missense | 32.0;  24.4 | Damaging | Not described |
|  | *ZFHX4* | Virus Interaction | NM_024721.5 | c.969G>C;  p.Gln323His | Missense | 25.9;  3.313 | Damaging | 0.000004016 |
|  | *FGL1* | Adaptive Immunity | NM_004467.4 | c.842A>T;  p.Asn281Ile | Missense | 26.2;  3.313 | Damaging | Not described |
|  | *BAG1* | Apoptosis | NM_004323.6 | c.929G>A;  p.Gly310Asp | Missense | 19.1;  3.313 | Tolerated | Not described |
|  | *RACGAP1* | Virus Interaction | NM_001319999.2 | c.205G>A;  p.Asp69Asn | Missense | 25.4;  3.313 | Damaging | 0.0005727 |
|  | *PIK3R6* | Innate Immunity | NM_001010855.4 | c.1177C>A;  p.Leu393Met | Missense | 24.5;  3.313 | N/S | 0.000004229 |
|  | *PTPN13* | Innate Immunity & Apoptosis | NM_080685.3 | c.6272-1G>A | Splice-Site | 33.0;  3.313 | N/S | Not described |
|  | *DERA* | Innate Immunity | NM_015954.4 | c.637+3_637+6delAAGT | Splice-Site | 35.0;  3.313 | N/S | Not described |
|  | *HTATSF1* | Virus Interaction | NM_014500.5 | c.794T>C;  p.Val265Ala | Missense | 26.5;  3.313 | Damaging | 0.000005593 |
|  | *ANAPC10* | Innate Immunity | NM_001256706.2 | c.527T>C;  Ile176Thr | Missense | 21.7;  21.226 | Tolerated | Not described |
| P5 | *PIK3R2* | Autophagy | NM_005027.4 | c.1958G>T;  p.Gly653Val | Missense | 27.6;  23.5 | Damaging | Not described |
|  | *OCIAD2* | Innate Immunity | NM_001014446.3 | c.304T>C;  p.Tyr102His | Missense | 24.3;  3.313 | Damaging | 0.0002354 |
|  | *STAB1* | Innate Immunity | NM_015136.3 | c.3094C>T;  p.Arg1032Cys | Missense | 24.2;  3.313 | Damaging | 0.0005381 |
|  | *TIAM1* | Adaptive Immunity | NM_001353694.2 | c.2108C>G;  p.Ser703Cys | Missense | 28.6;  3.3131 | Damaging | 0.0006688 |
|  | *SERPING1* | Innate Immunity | NM_000062.3 | c.1198C>T;  p.Arg400Cys | Missense | 23.0;  0.003 | Damaging | 0.00004772 |
|  | *FLCN* | Autophagy | NM_144997.7 | c.268G>T;  p.Ala90Ser | Missense | 21.1;  5.546 | Tolerated | 0.0003660 |
|  | *ITGB6* | Virus Interaction | NM_000888.5 | c.1175C>T;  p.Ala392Val | Missense | 24.1;  23.400 | Damaging | 0.00003184 |
|  | *ILRUN* | Innate Immunity | NM_024294.4 | c.745_765del;  p.Thr249_Asp255del | Inframe-Deletion | 21.8;  N/S | N/S | Not described |
|  | *LYST* | Innate & Adaptive Immunity | NM_000081.4 | c.10143+6T>G | Splice-Site | 21.8;  0.456 | N/S | 0.00004378 |
|  | *ACKR1* | Innate Immunity | NM_002036.4 | c.940_943dupACCC;  p.Leu315Hisfs*10 | Frameshift | 1.94;  N/S | N/S | Not described |
|  | *CPN1* | Innate Immunity | NM_001308.3 | c.272T>G;  p.Leu91Trp | Missense | 25.0;  3.069 | Damaging | Not described |
|  | EPHA5 | Adaptive Immunity | NM_001281766.3 | c.368C>T;  p.Ala123Val | Missense | 24.7;  3.313 | Damaging | 0.000007958 |
| P6 | *APPL2* | Innate Immunity | NM_018171.5 | c.1408C>T;  p.Arg470Cys | Missense | 33.0;  3.313 | Damaging | 0.0001870 |
|  | *WDFY1* | Innate Immunity | NM_020830.5 | c.1136G>A;  p.Gly379Glu | Missense | 26.3;  6.122 | Damaging | 0.0001752 |
|  | *ZFHX3* | Innate Immunity | NM_006885.4 | c.1565A>G;  p.Asp522Gly | Missense | 24.3;  3.313 | Damaging | 0.00005567 |
|  | *PRKD1* | Innate Immunity | NM_002742.3 | c.1271T>C;  p. Met424Thr | Missense | 23.9;  5.778 | Damaging | Not described |
|  | *ITLN1* | Innate Immunity | NM_017625.3 | c.454G>A;  p.Val152Met | Missense | 25.7;  3.313 | Damaging | 0.00005629 |
|  | *CPNE3* | Autophagy | NM_003909.5 | c.130G>A;  p.Glu44Lys | Missense | 33.0;  5.764 | Damaging | 0.000004053 |
| P7 | *PMM2* | Innate Immunity | NM_000303.3 | c.669C>A;  p.Asp223Glu | Missense | 23.9;  2.652 | Damaging | Not described |
|  | *RBBP8* | Adaptive Immunity | NM_002894.3 | c.248G>A;  p.Arg83Gln | Missense | 35.0;  3.143 | Damaging | 0.000007963 |
|  | *FNIP1* | Autophagy & Adaptive Immunity | NM_133372.3 | c.674T>G;  p.Leu225Arg | Missense | 27.6;  3.313 | Damaging | 0.000007965 |
|  | *PARP3* | Adaptive Immunity | NM_001003931.4 | c.1223G>A;  p.Arg408His | Missense | 25.6;  3,.313 | Damaging | 0.0002212 |
|  | *BDKRB1* | Virus Interaction | NM_000710.4 | c.122C>T;  p.Pro41Leu | Missense | 24.0;  3.313 | Damaging | 0.0006244 |
|  | *PIDD1* | Apoptosis | NM_145886.4 | c.2363C>T;  p.Thr788Met | Missense | 25.4;  N/S | Damaging | 0.0001294 |
|  | *SCNN1A* | Virus Interaction | NM_001038.6 | c.1793G>A;  p.Gly598Glu | Missense | 22.8;  1.766 | Tolerated | 0.000007980 |
|  | *ZFP36L2* | Innate Immunity | NM_006887.5 | c.1205delG;  p.Gly402Alafs*59 | Frameshift | 23.4;  3.313 | N/S | Not described |
|  | *GCA* | Innate Immunity & Autophagy | NM_012198.5 | c.27+5G>A | Splice-Site | 26.4;  3.313 | N/S | 0.000004020 |
| P8 | *FGF13* | Innate Immunity | NM_001139500.2 | c.103T>A;  p.Ser35Thr | Missense | 25.1;  N/S | Damaging | 0.000005646 |
|  | *HSF4* | Innate Immunity | NM_001538.4 | c.523C>G;  p.Arg175Gly | Missense | 2.,5;  7.173 | Damaging | 0.00002524 |
|  | *USP31* | Innate Immunity | NM_020718.4 | c.1744A>G;  p.Ser582Gly | Missense | 26.6;  3.313 | Damaging | Not described |
|  | *HERC2* | Innate & Adaptive Immunity & Autophagy | NM_004667.6 | c.845C>G;  p.Pro282Arg | Missense | 26.5;  3.313 | Damaging | Not described |
|  | *CTBP2* | Innate Immunity | NM_022802.3 | c.322C>T;  p.Arg108Trp | Missense | 24.7;  3.313 | Damaging | 0.00009547 |
|  | *PIK3C2G* | Autophagy | NM_001288772.2 | c.3536A>G;  p.Tyr1179Cys | Missense | 25.8;  3.313 | Damaging | 0.000004290 |
|  | *GBF1* | Autophagy | NM_001377137.1 | c.3407T>C;  p.Met1136Thr | Missense | 26.8;  3.313 | Damaging | 0.000007964 |
|  | *DLG5* | Innate Immunity | NM_004747.4 | c.5635G>C;  p.Glu1879Gln | Missense | 28.1;  3.313 | Damaging | Not described |
|  | *CHMP7* | Autophagy | NM_152272.5 | c.881G>A;  p.Arg294His | Missense | 27.9;  3.313 | Tolerated | 0.00001989 |
|  | *ZC3HAV1* | Virus Interaction | NM_020119.4 | c.1994-2A>T | Splice-Site | 33.0;  3.313 | N/S | Not described |
|  | *AKAP8* | Innate Immunity | NM_005858.4 | c.169_189del;  p.Ser57_Ala63del | In-Frame Deletion | 16.8;  3.313 | N/S | Not described |
|  | *SCRIB* | Innate & Adaptive Immunity | NM_182706.5 | c.2497_2499delGAT;  p.Asp833del | In-Frame Deletion | 21.6;  14.420 | N/S | Not described |
|  | *SLAMF8* | Adaptive Immunity | NM_020125.3 | c.211_212delCT;  p.Leu71Valfs*92 | Frameshift | 24.3;  3.313 | N/S | Not described |
|  | *FLNC* | Autophagy | NM_001458.5 | c.2416G>A;  p.Ala806Thr | Missense | 23.2;  0.001 | Tolerated | Not described |
| P9 | *CFHR5* | Innate Immunity | NM_030787.4 | c.1291T>G;  p.Cys431Gly | Missense | 22.7;  8.495 | Damaging | Not described |
|  | *BRCA2* | Adaptive Immunity & Autophagy | NM_000059.4 | c.9501+3A>T | Splice-Site | N/S;  0.001 | N/S | Not described |
|  | *SPG7* | Innate Immunity | NM_003119.2 | c.2191G>A;  p.Ala731Thr | Missense | 24.0;  0.001 | N/S | 0.0000319 |
|  | *STAM2* | Innate Immunity | NM_005843.6 | c.959T>C;  p.Leu320Ser | Missense | 24.9;  5.448 | Tolerated | Damaging |
|  | *IL22RA2* | Innate Immunity | NM_052962.2 | c.596A>G;  p.Tyr199Cys | Missense | 24.9;  5.448 | Damaging | 0.0000201 |
|  | *CFTR* | Innate Immunity | NM_000492.4 | c.2770G>A;  p.Asp924Asn | Missense | 31.0;  0.001 | Damaging | 0.0000676 |
|  | *ZP4* | Innate Immunity | NM_021186.5 | c.530delA;  p.Asn177Ilefs*9 | Frameshift | N/S;  3.313 | N/S | Not described |
|  | *TOP1* | Virus Interaction | NM_003286.4 | c.1951-17A>- | Splice-Site | N/S;  3.313 | N/S | 0.00000398 |
|  | *ZNF335* | Adaptive Immunity | NM_022095.4 | c.3298_3300delCTC;  p.Glu1100del | In-Frame Deletion | N/S;  3.313 | N/S | Not described |
|  | *MAP3K15* | Innate Immunity | NM_001001671.4 | c.3353_3355delTGT;  p.Asn1118del | In-Frame Deletion | N/S;  0.305 | N/S | Not described |
| P10 | *TNKS2* | Innate & Adaptive Immunity | NM_025235.4 | c.354T>A;  p.Asn118Lys | Missense | 24,6;  11.999 | Damaging | 0.0000716 |
|  | *CUEDC2* | Innate Immunity | NM_024040.3 | c.703A>G;  p.Met235Val | Missense | 16.74;  3.313 | Tolerated | 0.00002 |
|  | *EIF2AK4* | Virus Interaction | NM_001013703.4 | c.1661-18C>G | Splice-Site | N/S;  0.005 | N/S | 0.00000417 |
|  | *CLEC10A* | Innate & Adaptive Immunity | NM_182906.4 | c.82C>T;  p.GLN28X | Stop Gain | 36.0;  3.313 | N/S | 0.0002 |
|  | *MYO9B* | Adaptive Immunity | NM_004145.4 | c.2811+19C>T | Splice-Site | N/S;  3.313 | N/S | 0.0000296 |
|  | *TEC* | Innate & Adaptive Immunity | NM_003215.3 | c.1603G>C;  p.Glu535Gln | Missense | 29.3;  3.313 | Damaging | Not described |
|  | *DCST1* | Innate Immunity | NM_152494.4 | c.1074_1088del;  p.Val359_Val363del | In-Frame Deletion | N/S;  3.313 | N/S | 0.0006 |
|  | *HPGD* | Innate Immunity | NM_001256307.2 | c.120delAla;  p.Ala41Glnfs*31 | Frameshift | N/S;  16.370 | N/S | 0.0000517 |
| P11 | *PTPRS* | Innate Immunity | NM_002850.4 | c.2845C>T;  p.Arg949Cys | Missense | 27.2;  3.313 | N/S | 0.0002 |
|  | *MSH6* | Adaptive Immunity | NM_000179.3 | c.3940C>G;  p.Gln1314Gu | Missense | 21.9;  0.026 | Tolerated | 0.000004 |
|  | *GGT5* | Adaptive Immunity | NM_001099781.2 | c.184C>T;  p.Gln62X | Stop Gain | 37.0;  3.313 | N/S | 0.0000493 |
|  | *FZD1* | Innate & Adaptive Immunity | NM_003505.2 | c.173T>C;  p.Leu58Pro | Missense | 19.3;  3.313 | Tolerated | 0.0000304 |
|  | *HAX1* | Innate Immunity | NM_006118.4 | c.116_117insAGAAGG;  p.Gly41_Gly42insGluGly | In-Frame Insertion | N/S;  0.003 | N/S | 0.0004 |
|  | *STAP1* | Adaptive Immunity | NM_012108.4 | c.827-1G>- | Splice-Site | N/S;  23.000 | N/S | 0.0000207 |
|  | *FBXW7* | Innate Immunity | NM_033632.3 | c.349_351del;  p.Glu117del | In-Frame Deletion | N/S;  3.313 | N/S | 0.0002 |
| P12 | *IPO13* | Innate & Adaptive Immunity | NM_014652.4 | c.821+20C>T | Splice-Site | N/S;  3.313 | N/S | 0.0002 |
|  | *RNF187* | Innate Immunity | NM_001010858.3 | c.391-17C>A | Splice-Site | N/S;  9.592 | N/S | Not described |
|  | *MYOF* | Innate & Adaptive Immunity | NM_013451.4 | c.3214C>T;  p.Arg1072Cys | Missense | 32.0;  3.313 | Damaging | 0.0003 |
|  | *MPEG1* | Innate Immunity | NM_001039396.2 | c.404G>T;  p.Arg135Met | Missense | 22.7;  3.313 | Damaging | 0.0000682 |
|  | *KLRG1* | Adaptive Immunity | NM_001329099.2 | c.164T>C  p.Leu55Pro | Missense | 22.8;  3.838 | Damaging | 0.0004 |
|  | *LRP6* | Innate & Adaptive Immunity | NM_002336.3 | c.3773C>T;  p.Thr1258Met | Missense | 28.1;  15.970 | Damaging | 0.0000279 |
|  | *ITPR2* | Autophagy | NM_002223.4 | c.2639G>A;  p.Ser880Asn | Missense | 20.6;  5.744 | Tolerated | 0.00000935 |
|  | *MMP14* | Virus Interaction | NM_004995.4 | c.1403T>C;  p.Met468Thr | Missense | 23.3;  5.744 | Tolerated | Not described |
|  | *SOS2* | Innate & Adaptive Immunity | NM_006939.4 | c.374A>T;  p.His125Leu | Missense | 22.5;  3.313 | Damaging | 0.0000249 |
|  | *BANP* | Adaptive Immunity | NM_017869.4 | c.673C>G;  p.Leu225Val | Missense | 25.3;  3.313 | Damaging | Not described |
|  | *COLEC11* | Innate Immunity | NM_024027.5 | c.434G>T;  p.Gly145Val | Missense | 23.8;  0.062 | Damaging | 0.0000519 |
|  | *TBC1D5* | Autophagy | NM_001134381.1 | c.1819-20T>C | Splice-Site | N/S;  3.313 | N/S | 0.0000364 |
|  | *EPHA5* | Adaptive Immunity | NM_004439.8 | c.1924+20T>A | Splice-Site | N/S;  3.313 | N/S | 0.0003 |
|  | *MYOCD* | Innate Immunity | NM_001146312.3 | c.2771_2772insTGGG;  p.Leu926Trpfs*8 | Frameshift | N/S;  3.313 | N/S | Not described |
|  | *PDLIM7* | Innate Immunity | NM_005451.5 | c.1319_1321delAGA;  p.Phe440del | In-Frame Deletion | N/S;  5.392 | N/S | 0.0000201 |
| P13 | *MACF1* | Innate Immunity | NM_012090.5 | c.14591A>G;  p.Lys4864Arg | Missense | 29.3;  3.313 | Tolerated | 0.00000398 |
|  | *DMAP1* | Innate Immunity | NM_019100.5 | c.848G>A;  p.Arg283His | Missense | 31.0;  3.313 | Tolerated | 0.0003 |
|  | *DRAM2* | Autophagy | NM_178454.6 | c.737T>C;  p.Leu246Pro | Missense | 28.6;  3.313 | Damaging | 0.0003 |
|  | *PTPN22* | Innate & Adaptive Immunity | NM_015967.8 | c.684-20A>G | Splice-Site | N/S;  3.313 | N/S | Not described |
|  | *CD244* | Innate & Adaptive Immunity | NM_016382.4 | c.61+18G>C | Splice-Site | N/S;  3.313 | N/S | 0.0001 |
|  | *CAPN2* | Innate Immunity | NM_001748.5 | c.281G>A;  p.Arg94His | Missense | 32.0;  3.313 | Damaging | 0.0000279 |
|  | *DNA2* | Innate Immunity | NM_001080449.3 | c.239G>T;  p.Cys80Phe | Missense | 28.6;  0.204 | Damaging | Not described |
|  | *RAC2* | Adaptive Immunity | NM_002872.5 | c.40G>A;  p.Val14Met | Missense | 32.0;  25.874 | Damaging | Not described |
|  | *PLA2G6* | Innate Immunity | NM_003560.4 | c.1424G>A;  p.Arg475Gln | Missense | 23.5;  1.955 | Tolerated | 0.0002 |
|  | *PLD1* | Innate & Adaptive Immunity | NM_002662.5 | c.665+1G>A | Splice-Site | 34.0;  3.313 | N/S | 0.0000478 |
|  | *HTR4* | Innate & Adaptive Immunity | NM_001040169.2 | c.691C>T;  p.Arg231Trp | Missense | 24.1;  3.313 | Damaging | 0.0001 |
|  | *EEF1D* | Virus Interaction | NM_032378.7 | c.1488+17C>T | Splice-Site | N/S;  3.313 | N/S | 0.0000802 |
|  | *RASAL3* | Adaptive Immunity | NM_022904.3 | c.2455_2456del;  p.Ser819Cysfs*84 | Frameshift | N/S;  3.313 | N/S | 0.0000132 |
|  | *TWIST1* | Innate Immunity | NM_000474.4 | UTR3 | Splice-Site | N/S;  6.18 | N/S | Not described |
|  | ZFHX4 | Virus Interaction | NM_024721.5 | c.8847T>G;  p.Ser2949Arg | Missense | 16.0;  3.313 | Damaging | 0.00000803 |
| P14 | *UBR4* | Innate Immunity | NM_020765.3 | c.1709A>G;  p.Tyr570Cys | Missense | 27.5;  3.313 | Damaging | Not described |
|  | *DDX51* | Innate & Adaptive Immunity | NM_175066.4 | c.995+19C>T | Splice-Site | N/S;  3.313 | N/S | 0.0000363 |
|  | *TRPC3* | Adaptive Immunity | NM_001130698.2 | c.2547+3A>G | Splice-Site | N/S;  3.313 | N/S | 0.000056 |
| P15 | *SLC7A1* | Adaptive Immunity & Virus Interaction | NM_003045.5 | c.1084A>G;  p.Ile362Val | Missense | 23.5;  5.744 | Tolerated | 0.00000398 |
|  | *HK3* | Innate & Adaptive Immunity | NM_002115.3 | c.1903G>A;  p.Glu635Lys | Missense | 26.2;  3.313 | Tolerated | 0.0001 |
|  | *MTMR9* | Autophagy & Apoptosis | NM_015458.4 | c.1108C>A;  p.Leu370Met | Missense | 25.3;  3.313 | Damaging | 0.0002 |
|  | *WRN* | Innate Immunity | NM_000553.6 | c.130C>G;  p.Leu44Val | Missense | 20.7;  0.001 | Damaging | 0.0004 |
|  | *NSF* | Autophagy | NM_006178.4 | c.2111_2113delGGA;  p.Trp704_Ile705delinsLeu | In-Frame Deletion | N/S;  17.812 | N/S | Not described |
|  | *NSF* | Autophagy | NM_006178.4 | c.2115_2126deAGGAATCAAGAAl;  p.Ile705_Lys709delinsMet | In-Frame Deletion | N/S;  17.812 | N/S | Not described |
|  | *BPIFA1* | Innate Immunity | NM_016583.4 | c.586delG;  p.Gly196Alafs*16 | Frameshift | N/S;  3.313 | N/S | 0.00000398 |
| P16 | *TIGAR* | Innate Immunity | NM_020375.3 | c.124G>A;  p.Ala42Thr | Missense | 21.5;  N/S | Damaging | Not described |
|  | *RGMA* | Innate Immunity | NM_001166283.2 | c.578C>T;  p.Ala193Val | Missense | 21.3;  3.313 | Tolerated | 0.0000213 |
|  | *TNFSF12* | Innate Immunity & Apoptosis | NM_003809.3 | c.353G>A;  p.Gly118Glu | Missense | 27.2;  8.923 | Damaging | 0.0002 |
|  | *TNFSF12-TNFSF13* | Innate Immunity | NM_172089.4 | c.353G>A;  p.Gly118Glu | Missense | N/S;  N/S | N/S | 0.0002 |
|  | *DNAJC27* | Innate Immunity | NM_016544.3 | c.171-20T>G | Splice-Site | 20.2;  5.778 | Damaging | 0.0001 |
|  | *AGA* | Virus Interaction | NM_000027.4 | c.481C>T;  p.Arg161Trp | Missense | N/S;  3.940 | N/S | 0.00000901 |
|  | *PTGES2* | Innate Immunity | NM_025072.7 | c.1006-20C>T | Splice-Site | N/S;  3.313 | N/S | 0.00000948 |
| P17 | *TP73* | Apoptosis | NM_005427.4 | c.1074+19A>G | Splice-Site | N/S;  3.313 | N/S | Not described |
|  | *NASP* | Innate Immunity | NM_002482.4 | c.1593-18C>A | Splice-Site Variant | N/S;  3.313 | N/S | 0.0000756 |
|  | *SLC18A2* | Adaptive Immunity | NM_003054.6 | c.127A>G;  p.Ile43Val | Missense | 24.4;  3.313 | Damaging | 0.00000402 |
|  | *NR1H3* | Innate Immunity | NM_005693.4 | c.931G>A;  p.Glu311Lys | Missense | 26.1;  3.313 | Tolerated | 0.00000398 |
|  | *APAF1* | Adaptive Immunity & Apoptosis | NM_013229.3 | c.139-17T>C | Splice-Site | N/S;  17.650 | N/S | Not described |
|  | *POLE* | Adaptive Immunity | NM_006231.4 | c.2773T>C;  p.Ser925Pro | Missense | 24.0;  12.420 | Damaging | 0.0000557 |
|  | *CCDC177* | Virus Interaction | NM_001271507.2 | c.1495C>T;  p.Arg499Trp | Missense | 24.9;  3.313 | N/S | Not described |
|  | *TRAF7* | Innate Immunity | NM_032271.3 | c.421C>G;  p.Pro141Ala | Missense | 23.2;  5.751 | Damaging | Not described |
|  | *PIK3R5* | Autophagy | NM_001142633.3 | c.2577G>C;  p.Gln859His | Missense | 21.4;  3.313 | Damaging | Not described |
|  | *MED16* | Innate Immunity | NM_005481.3 | c.2101C>T;  p.Arg701Cys | Missense | 25.0;  3.313 | Damaging | 0.0003 |
|  | *GRAMD1A* | Autophagy | NM_020895.5 | c.1512G>T;  p.Trp504Cys | Missense | 32.0;  3.313 | Damaging | Not described |
|  | *RIF1* | Adaptive Immunity | NM_018151.5 | c.7095+18A>G | Splice-Site | N/S;  34.000 | N/S | 0.00000437 |
|  | *HOXD11* | Autophagy | NM_021192.3 | c.1A>T;  p.Met1? | Start Loss | 25.4;  7.290 | Damaging | 0.0000943 |
|  | *ARHGEF3* | Autophagy | NM_001128615.2 | c.301-17T>C | Splice-Site Variant | N/S;  3.313 | N/S | 0.00000809 |
|  | *ATP6V1A* | Virus Interaction | NM_001690.4 | c.1688A>G;  p.Asn563Ser |  | 19.8;  4.946 | Tolerated | 0.0000438 |
|  | *RPA3* | Autophagy | NM_002947.5 | c.99+16A>G | Splice-Site | N/S;  5.374 | N/S | 0.0002 |
|  | *GLI3* | Adaptive Immunity & Autophagy | NM_000168.6 | c.1813-20G>A | Splice-Site | N/S;  5.176 | N/S | 0.0000208 |
|  | *FLNC* | Autophagy | NM_001458.5 | c.5311C>G;  p.Pro1771Ala | Missense | 22.7;  0.001 | Tolerated | 0.0002 |
|  | *MUC1* | Innate & Adaptive Immunity | NM_002456.6 | c.516_534del GCCCCAGCCTGG CACCCCA;  p.Gly173Serfs*46 | Frameshift | N/S;  3.313 | N/S | 0.00000401 |
|  | *LTK* | Innate Immunity | NM_002344.6 | c.401delA;  p.Asn134Thrfs*107 | Frameshift | N/S;  3.313 | N/S | 0.0003 |
|  | *TRNT1* | Autophagy | NM_182916.3 | c.126_128del;  p.Glu43del | In-Frame Deletion | N/S;  1.812 | N/S | 0.0000318 |
|  | *ERAP2* | Adaptive Immunity | NM_022350.5 | c.151delG;  p.Ala52Leufs*4 | Frameshift | N/S;  3.313 | N/S | Not described |

Variants identified in patients with ARN. Abbreviations: P, patient; ID, identification; ARN, acute retinal necrosis, N/S, not specified; CADD, combined annotation dependent depletion; MSC, mutation significance cutoff; SIFT, Sorting Intolerant from Tolerant; gnomAD, genome aggregation database.

## **Supplementary Table 2.** **Variants identified in the patient cohort without substantial known role in immunity, autophagy or apoptosis.**

| P ID | Gene Symbol | Transcript ID | Transcript Variant; Protein Variant | Translation Impact | CADD; MSC Scores | SIFT Function Prediction | GnomAD Frequency (%) |
| --- | --- | --- | --- | --- | --- | --- | --- |
| P1 | *RCBTB2* | NM_001268.4 | c.142G>C;  p.Ala48Pro | Missense | 27.9; 3.313 | Damaging | 0.00008136 |
|  | *E2F6* | NM_198256.4 | c.565C>T;  p.His189Tyr | Missense | 22.8; 3.313 | Damaging | 0.00004415 |
|  | *SEMA4G* | NM_017893.4 | c.2068G>A;  p.Gly690Ser | Missense | 24.1; 3.313 | Damaging | 0.0006673 |
|  | *HEXB* | NM_000521.4 | c.1199A>G;  p.Lys400Arg | Missense | 23.0; 0.004 | Tolerated | 0.00003184 |
|  | *RBM26* | NM_001366735.2 | c.2912_2914delAAG; p.Glu971del | In-Frame Deletion | 21.9; 5.771 | N/S | 0.000007985 |
| P2 | *DYSF* | NM_003494.4 | c.244G>A;  p.Gly82Arg | Missense | 27.9; 0.009 | Damaging | 0.00001989 |
|  | *TLX1* | NM_005521.4 | c.431T>G;  p.Val144Gly | Missense | 19.9; 3.313 | Tolerated | Not described |
|  | *LNPK* | NM_030650.3 | c.224C>T;  p.Ala75Val | Missense | 16.6; N/S | Tolerated | 0.0001503 |
|  | *SRRM5* | NM_001145641.2 | c.352delC; p.His118Thrfs*28 | Frameshift | 20.3; 3.313 | N/S | Not described |
|  | *GALC* | NM_000153.4 | c.74_75insGGGGGGGC; p.Arg26Glyfs*49 | Frameshift | 23.3; 8.428 | N/S | Not described |
|  | *CNOT6* | NM_001370472.1 | c.718-5T>C | Splice-Site | 16.7; 3.313 | N/S | 0.00004218 |
|  | *AOX1* | NM_001159.4 | c.2655+3_2655+6delAAGT | Splice-Site | 32.0; 3.313 | N/S | Not described |
|  | *RECQL5* | NM_004259.7 | c.1504C>T;  p.Arg502Trp | Missense | 28.7; 3.313 | Damaging | Not described |
| P3 | *NUP205* | NM_015135.3 | c.2182C>T;  p.Arg728Trp | Missense | 28.6; 5.785 | Damaging | Not described |
|  | *ABCB4* | NM_000443.4 | c.3296A>G;  p.Glu1099Gly | Missense | 27.3;  0.009 | Damaging | 0.0004061 |
|  | *SYNJ2* | NM_003898.4 | c.3556G>A;  p.Ala1186Thr | Missense | 25.6;  3.313 | Damaging | 0.0004024 |
|  | *PAICS* | NM_001079524.2 | c.158A>G;  p.Lys53Arg | Missense | 24.1;  3.313 | Damaging | 0.0007931 |
|  | *MRPS17* | NM_015969.3 | c.121A>C;  p.Lys41Gln | Missense | 24.3;  3.313 | Tolerated | Not described |
|  | *CAD* | NM_004341.5 | c.5444G>T;  p.Gly1815Val | Missense | 21.3;  6.004 | Tolerated | 0.00002564 |
|  | *MYO1C* | NM_033375.5 | c.1611+2T>G | Splice-Site | 33.0;  5.516 | N/S | Not described |
| P4 | *MYO1F* | NM_012335.4 | c.1867T>C;  p.Tyr623His | Missense | 28.0;  3.313 | Damaging | 0.00001205 |
|  | *KLKB1* | NM_000892.5 | c.1561G>A;  p.Gly521Arg | Missense | 25.2;  25.800 | Damaging | 0.0001353 |
|  | *CRYAA* | NM_000394.4 | c.275A>G;  p.Asp92Gly | Missense | 24.1;  14.420 | Damaging | 0.000003986 |
|  | *BLMH* | NM_000386.4 | c.653G>A;  p.Arg218Gln | Missense | 32.0;  7.253 | Damaging | 0.00001993 |
|  | *MYH2* | NM_017534.6 | c.5122G>A;  p.Ala1708Thr | Missense | 24.3;  0.003 | Damaging | 0.00009947 |
| P5 | *CNKSR2* | NM_014927.5 | c.857C>T;  p.Pro286Leu | Missense | 26.0;  5.764 | Damaging | 0.000005484 |
| P6 | *AGL* | NM_000642.3 | c.1028G>A;  p.Arg343Gln | Missense | 25.2;  0.021 | Damaging | 0.0004218 |
|  | *BMP1* | NM_006129.5 | c.2498C>T;  p.Thr833Ile | Missense | 26.3;  19.040 | Damaging | 0.000004006 |
|  | *GPR146* | NM_001303473.2 | c.392G>A;  p.Arg131His | Missense | 23.9;  3.313 | Damaging | 0.000004046 |
|  | *GPR17* | NM_001161417.2 | c.914G>A;  p.Arg305His | Missense | 29.8;  3.313 | Damaging | 0.0006294 |
|  | *VTI1B* | NM_006370.3 | c.146A>G;  p.Asp49Gly | Missense | 23.9;  3.313 | Damaging | Not described |
|  | *COL28A1* | NM_001037763.3 | c.2589G>C;  p.Leu863Phe | Missense | 23.9;  3.313 | Tolerated | Not described |
| P7 | *CENPJ* | NM_018451.5 | c.3589G>A;  p.Val1197Met | Missense | 22.9;  10.020 | Damaging | 0.00009943 |
|  | *CHD7* | NM_017780.4 | c.3347A>G;  p.Lys1116Arg | Missense | 28.5;  0.019 | Damaging | 0.000004018 |
|  | *CLPTM1L* | NM_030782.5 | c.1246G>C;  p.Ala416Pro | Missense | 23.8;  3.313 | Damaging | 0.000003979 |
|  | *PRSS27* | NM_031948.5 | c.293C>T;  p.Pro98Leu | Missense | 23.7;  3.313 | Tolerated | 0.0005244 |
|  | *MGA* | NM_001164273.2 | c.8126G>T;  p.Gly2709Val | Missense | 22.5;  3.313 | Tolerated | 0.00005225 |
|  | *DDR1* | NM_001297654.2 | c.2695delT;  p.Ser899Profs | Missense | 29.4;  5.708 | N/S | Not described |
| P8 | *PTPN3* | NM_002829.4 | c.650A>G;  p.Tyr217Cys | Missense | 27.3;  3.313 | Damaging | 0.00001194 |
|  | *EVI2A* | NM_014210.4 | c.39T>A;  p.His13Gln | Missense | 21.4;  3.313 | Damaging | Not described |
|  | *GPNMB* | NM_001005340.2 | c.1574A>C;  p.Tyr525Ser | Missense | 25.4;  3.313 | Damaging | 0.00004406 |
|  | *EP400* | NM_015409.5 | c.7703C>T;  p.Thr2568Met | Missense | 22.5;  3.313 | Damaging | 0.00005341 |
|  | *PRDM4* | NM_012406.4 | c.484A>G;  p.Ile162Val | Missense | 22.7;  3.313 | Damaging | 0.000003976 |
|  | *CEP290* | NM_025114.4 | c.2090C>G;  p.Ala697Gly | Missense | 25.2;  2.165 | Damaging | 0.0003743 |
|  | *RSF1* | NM_016578.4 | c.1819C>G;  p.Pro607Ala | Missense | 20.5;  3.313 | Damaging | 0.00008000 |
|  | *PPM1J* | NM_005167.7 | c.1409G>A;  p.Arg470Gln | Missense | 32.0;  3.313 | Tolerated | 0.00006654 |
|  | *MNT* | NM_020310.3 | c.73+1G>C | Splice-Site | 35.0;  3.313 | N/S | Not described |
|  | *NELFB* | NM_015456.5 | c.28C>T;  p.Arg10Trp | Missense | 17.4;  3.313 | N/S | Not described |
|  | *SMC2* | NM_006444.3 | c.2118_2120delAAA;  p.Lys706del | In-Frame Deletion | 21.1;  5.324 | N/S | Not described |
|  | *ODF1* | NM_024410.4 | c.673_683delAACCCGTGCAG;  p.Asn225Profs*3 | Frameshift | 35.0  3.313 | N/S | Not described |
|  | *ARID4B* | NM_016374.6 | c.666-3_666-2delTA | Splice-Site | 23.4;  3.313 | N/S | Not described |
| P9 | *CDC42BPA* | NM_014826.4 | c.4034G>A;  p.Arg1345Gln | Missense | 29.1;  3.313 | Damaging | 0.0000122 |
| P10 | *ZSCAN29* | NM_152455.4 | c.929G>A;  p.Arg310Gln | Missense | 28.0;  3.313 | Damaging | 0.0000597 |
|  | *RPL27* | NM_000988.5 | 5´ UTR | Splice-Site | N/S;  N/S | N/S | Not described |
|  | *ZNF512* | NM_032434.3 | c.373+20A>G | Splice-Site | N/S;  5.798 | N/S | 0.000024 |
|  | *KMT5B* | NM_017635.5 | c.2490_2492delGAG;  p.Ser832del | In-Frame Deletion | N/S;  N/S | N/S | 0.0000559 |
| P11 | *VPS72* | NM_001271087.2 | c.266A>G;  p.Tyr89Cys | Missense | 29.6;  3.313 | Damaging | 0.0002 |
|  | *CDC42BPA* | NM_014826.5 | c.4712C>T;  p.Pro1571Leu | Missense | 24.5;  3.313 | Tolerated | 0.00000398 |
|  | *CDC42BPG* | NM_017525.3 | c.4229T>G;  p.Phe1410Cys | Missense | 26.0;  3.313 | Damaging | 0.0000159 |
|  | *TCN2* | NM_000355.4 | c.449C>T;  p.Pro150Leu | Missense | 23.2;  0.001 | Damaging | N/S |
| P12 | *MYH2* | NM_017534.6 | c.193G>C;  p.Glu65Gln | Missense | 22.5;  0.003 | Tolerated | 0.0000358 |
| P13 | *CYFIP1* | NM_014608.6 | c.2159+10T>C | Splice-Site | N/S;  5.764 | N/S | 0.0000517 |
|  | *CAD* | NM_004341.5 | c.5147C>T;  p.Thr1716Met | Missense | 26.1;  6.004 | Damaging | 0.000056 |
|  | *CAD* | NM_004341.5 | c.5561G>A;  p.Arg1854Gln | Missense | 24.2;  6.004 | Tolerated | 0.0000437 |
|  | *ESRRB* | NM_004452.4 | c.602delT;  p.Ser202Lfs*8 | Frameshift | N/S;  11.410 | N/S | Not described |
| P14 | *TUBAL3* | NM_024803.2 | c.245T>C;  p.Ile82Thr | Missense | 18.8;  3.313 | Damaging | 0.0000677 |
|  | *MYO9A* | NM_024803.3 | c.511A>G;  p.Lys171Glu | Missense | 21.3;  3.313 | Tolerated | Not described |
|  | *ERCC4* | NM_005236.3 | c.2395C>T;  p.Arg799Trp | Missense | 31.0;  0.001 | Damaging | 0.0005 |
|  | *TRPV6* | NM_018646.6 | spl | Splice-Site | N/S;  3.313 | N/S | Not described |
| P15 | *SREK1* | NM_001077199.3 | c.847C>T;  p.Arg283X | Stop Gain | 37.0;  5.256 | N/S | Not described |
| P16 | *DENND4C* | NM_017925.7 | c.4634+7C>A | Splice-Site | N/S;  3.313 | N/S | 0.0000419 |
| P17 | *MYO5B* | NM_001080467.3 | c.4240G>A;  p.Glu1414Lys | Missense | 32.0;  0.017 | Damaging | 0.0004 |
|  | *MYH9* | NM_002473.6 | c.5026A>G;  p.Lys1676Glu | Missense | 25.4;  5.427 | Damaging | 0.0003 |
|  | *ANKRD28* | NM_015199.4 | c.574G>A;  p.Ala192Thr | Missense | 28.1;  5.751 | Damaging | 0.0003 |
|  | *RAD50* | NM_005732.4 | c.353delT;  p.Thr119Leufs*11 | Frameshift | N/S;  0.001 | N/S | 0.000012 |

Variants identified in patients with ARN. Abbreviations: P, patient; ID, identification; ARN, acute retinal necrosis, N/S, not specified; CADD, combined annotation dependent depletion; MSC, mutation significance cutoff; SIFT, Sorting Intolerant from Tolerant; gnomAD, genome aggregation database.
